# Identification of a staphylococcal dipeptidase involved in the production of human body odor

**DOI:** 10.1016/j.jbc.2024.107928

**Published:** 2024-10-24

**Authors:** Reyme Herman, Bethan Kinniment-Williams, Michelle Rudden, Alexander Gordon James, Anthony J. Wilkinson, Barry Murphy, Gavin H. Thomas

**Affiliations:** 1 Department of Biology, University of York, York, UK; 2 York Biomedical Research Institute, University of York, York, UK; 3 Hull York Medical School, University of York, York, UK; 4 School of Life Sciences, University of Hull, Hull, UK; 5 Unilever, Colworth Science Park, Bedfordshire, UK; 6 York Structural Biology Laboratory, Department of Chemistry, University of York, York, UK; 7 Unilever Research & Development, Port Sunlight Laboratory, Merseyside, UK

**Keywords:** peptidase, Cys-Gly-3M3SH, peptides, metalloenzyme, enzyme kinetics, enzyme structure, malodor, *Staphylococcus hominis*

## Abstract

The production of human body odor is the result of the action of commensal skin bacteria, including *Staphylococcus hominis*, acting to biotransform odorless apocrine gland secretions into volatile chemicals like thioalcohols such as 3-methyl-3-sulphanylhexan-1-ol (3M3SH). As the secreted odor precursor Cys-Gly-3M3SH contains a dipeptide, yet the final enzyme in the biotransformation pathway only functions on Cys-3M3SH, we sought to identify the remaining step in this human-adapted biochemical pathway using a novel coupled enzyme assay. Purification of this activity from *S. hominis* extracts led to the identification of the M20A-family PepV peptidase (ShPepV) as the primary Cys-Gly-3M3SH dipeptidase. To establish whether this was a primary substrate for PepV, the recombinant protein was purified and demonstrated broad activity against diverse dipeptides. The binding site for Cys-Gly-3M3SH was predicted using modeling, which suggested mutations that might accommodate this ligand more favorably. Indeed, a D437A resulted in an almost sixfold increase in the *k*_cat_*/K*_*m*_, whereas other introduced mutations reduced or abolished function. Together, these data identify an enzyme capable of catalyzing the missing step in an ancient human-specific biochemical transformation and suggest that the production of 3M3SH uses neither a dedicated transporter nor a peptidase for its breakdown, with only the final cleavage step, catalyzed by PatB cysteine-S-conjugate β-lyase, being a unique enzyme.

The production of body odor or malodor in humans is a process in which microbes on the skin play a central role through their bioconversion of odorless secreted molecules to volatile odorants (1, 2, 3). These precursor molecules are produced by the apocrine glands, which are associated exclusively with hair follicles in specific sites like the underarm and the genital regions. Unlike eccrine sweat glands that are found in all human skin and have roles in thermoregulation, the physiological functions of apocrine gland secretions are less well understood (4). However, it is known that human malodor precursors like S-(1-hydroxy-3-methylhexan-3-yl)-l-cysteinylglycine (Cys-Gly-3M3SH) (5), volatile fatty acid–conjugated glutamine (6, 7), steroids, and long-chain fatty acids (8, 9) are secreted onto the human skin *via* the apocrine gland, and there is now extensive evidence that many of these compounds are immediately metabolized by the resident skin microbiome living within the hair follicle or the surrounding surface (9, 10).

The human skin is stably colonized by a unique community of microbes collectively known as the skin microbiome. Taxonomic characterization of the human skin microbiome (11, 12) has identified *Staphylococcus* as key genera that persistently colonize the skin, alongside *Cutibacterium* and *Corynebacteria* species. The axilla hosts its own unique microbiota (13) which is dominated by both staphylococci and corynebacteria (14). Both these microbial genera play a key role in axillary malodor formation. Bacterial biotransformation of the odorless Cys-Gly-3M3SH precursor involves its uptake *via* a peptide transporter and subsequent enzymatic action by a peptidase followed by a cysteine-S-conjugate β-lyase (C-S β-lyase), which generates the highly pungent volatile molecule 3M3SH (15, 16, 17, 18, 19) (Fig. 1*A*). Initially, the dipeptidase and the C-S β-lyase capable of the complete enzymatic processing of Cys-Gly-3M3SH were identified in an axillary isolate of corynebacteria (17, 18). Subsequently, work from us and others focused on the more predominant staphylococci and identified key roles for specific *Staphylococcal* species in 3M3SH production using whole-cell *in vivo* assays with the physiological Cys-Gly-3M3SH precursor. We identified a host-restricted clade of axillary staphylococci including *Staphylococcus hominis* and *Staphylococcus hemolyticus* (5, 16, 20) as the key microbial species capable of metabolizing Cys-Gly-3M3SH directly to volatile 3M3SH. We identified the molecular basis for the biotransformation in *S. hominis* for two of the three steps (1): a proton-dependent oligopeptide transporter required for uptake of Cys-Gly-3M3SH (15) and (2) a uniquely modified pyridoxal 5′-phosphate–dependent PatB enzyme that cleaves Cys-3M3SH leading to the production of 3M3SH (16). Importantly, this enzyme specifically recognizes the Cys-3M3SH precursor and not the dipeptide-conjugated Cys-Gly-3M3SH, suggesting that an intracellular staphylococcal dipeptidase is essential for production of human malodor.

**Figure 1:**
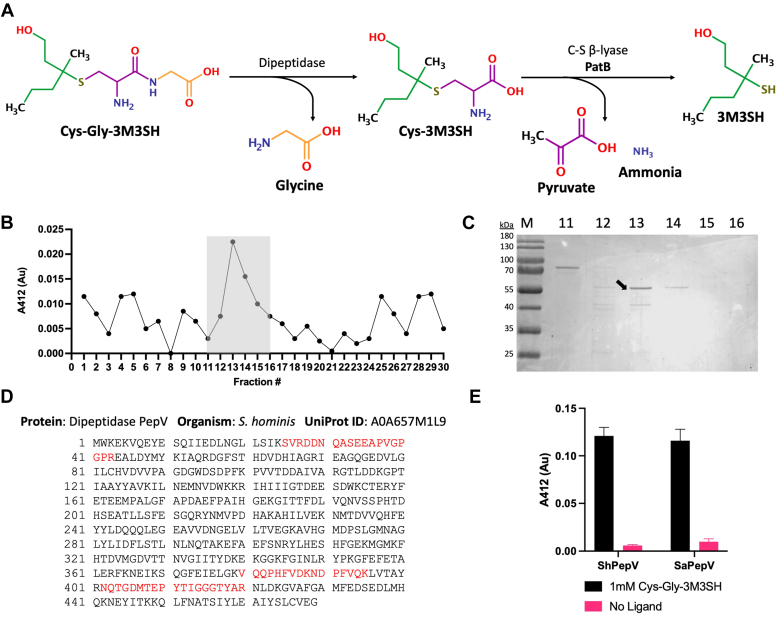
**Biochemical identification of PepV as a peptidase active on Cys-Gly-3M3SH.***A*, the generation of the odorous molecule 3M3SH from Cys-Gly-3M3SH in *Staphylococcus hominis* is catalyzed by a dipeptidase and PatB, a C-S β-lyase. *B* and *C*, isolation and (*D*) identification of the dipeptidase ShPepV, a protein (*E*) capable of Cys-Gly-3M3SH dipeptidase activity in *S. hominis*. A coupled DTNB assay was used to colorimetrically detect Cys-Gly-3M3SH dipeptidase function in ShPepV and *Staphylococcus aureus* PepV (SaPepV). *B*, *shaded region* indicates the fractions further analyzed. *C*, SDS-PAGE analysis of column fractions 11–16—*arrow* denotes the protein band excised for analysis by mass spectrometry. *D*, sequence of PepV from *S. hominis*—the segments in *red* denote peptides that were identified by mass spectrometry. *E*, results of coupled DTNB assays suggest the abilities of both ShPepV and SaPepV in contributing to the biotransformation of Cys-Gly-3M3SH (data in technical triplicates with errors [SD]). C-S β-lyase, cysteine-S-conjugate β-lyase; DTNB, 5,5′-dithiobis-(2-nitrobenzoic acid); 3M3SH, 3-methyl-3-sulphanylhexan-1-ol.

Through a biochemical approach, the Cys-Gly-3M3SH active corynebacterial M20A family dipeptidase TpdA was identified in the skin isolate *Corynebacterium* sp. Ax20 (17). This protein, which is thought to be a metallopeptidase of the succinyldiaminopimelate desuccinylase (DapE) subfamily, was found to be active against Cys-Gly-3M3SH but not against the nonconjugated dipeptide of Cys-Gly. Here, we set out to identify the dipeptidase responsible for this function in the staphylococcal malodor producer, *S. hominis*. We report the identification and biochemical characterization of the Cys-Gly-3M3SH active promiscuous metallopeptidase PepV from *S. hominis*.

## Results

### Biochemical identification of a dipeptidase with activity against Cys-Gly-3M3SH

To identify the enzyme(s) catalyzing the conversion of Cys-Gly-3M3SH to Cys-3M3SH in staphylococci, we first looked for homologs of the *Corynebacterium* M20-family dipeptidase TpdA, which is known to catalyze this reaction (17) but no homologs were present in any cutaneous *Staphylococcus* genomes. Therefore, we widened our computational analysis of putative peptidases using the MEROPS database (21), which predicted 40 such enzymes encoded in *S. hominis*, 10 of which were classified to be of the M20 family that contains TpdA. Previously reported examples include the tripeptidase PepT (22, 23) of the M20B subfamily and the promiscuous dipeptidase PepV (24, 25) of the M20A subfamily. Alternatively, the M17 subfamily leucyl aminopeptidase PepA might also possess Cys-Gly dipeptidase activity with previous homologs shown to have this activity (26, 27, 28). Because of the high number of potential enzymes and the lack of genetic tools to manipulate *S. hominis*, a biochemical approach was undertaken to isolate the major Cys-Gly-3M3SH dipeptidase from cell extracts.

To enable us to screen for the desired activity in cellular fractions, we modified a 5,5′-dithiobis-(2-nitrobenzoic acid) (DTNB) assay that detects 3M3SH, which we used previously to study the function of the C-S β-lyase ShPatB *in vitro* (16) and the transporter DtpT *in vivo* (15). Here, we exploit the fact the ShPatB will not work on the substrate of the dipeptidase reaction, Cys-Gly-3M3SH, but will act on its product, Cys-3M3SH, to produce the DTNB-reactive 3M3SH (Data S1). Whole-cell extracts of *S. hominis* B10 were prepared and fractionated using a series of chromatographic methods using the coupled ShPatB-DTNB assay to measure biochemical activity across fractions. Proteins were first separated on a strong anion exchange (AEX) column followed by a hydrophobic interactions chromatography column, then a weak AEX column, and finally by size-exclusion chromatography (Datas S2–S5). Following the final step, fractions with detectable activity were pooled (Fig. 1*B*), concentrated, and analyzed by SDS-PAGE revealing a prominent species with an apparent molecular mass of ∼55 kDa in Fraction 13 (Fig. 1*C* and Data S6). This band was excised, and the protein within it was unambiguously determined to be the PepV protein from *S. hominis* by peptide mass fingerprinting (ShPepV) through the identification of three distinct and one modified peptide (Fig. 1*D* and Data S7).

To confirm that ShPepV has dipeptidase activity against Cys-Gly-3M3SH, we recombinantly expressed and purified ShPepV and the structurally characterized *Staphylococcus aureus* homolog SaPepV (29). Using the coupled DTNB assay, it was determined that both enzymes, when present at 1 μM, had activity on 1 mM Cys-Gly-3M3SH (Fig. 1*E*). These data suggest that PepV is the major peptidase catalyzing this reaction in extracts of *S. hominis*. Since PepV from nonthioalcohol producing *S. aureus* (which lacks the C-S lyase PatB) can also catalyze this reaction, we suggest that PepV dipeptidase activity is most likely conserved among other staphylococci and has not specifically evolved for malodor biotransformation.

### ShPepV is a general dipeptidase active on diverse L-amino acid–containing dipeptides

Having established that ShPepV catalyzes the production of Cys-3M3SH from Cys-Gly-3M3SH, and that the corresponding enzyme from a nonthioalcohol-producing staphylococci also catalyzes this reaction, we tested the hypothesis that this protein has a more general cellular function in dipeptide turnover, rather than a specialized role in malodor production. Indeed, SaPepV is known to cleave various dipeptides and also to hydrolyze β-lactams (30). Similar activity has been observed for other bacterial M20A enzymes such as the PepV from *Lactobacillus delbrueckii* subsp. *lactis* DSM 7290 (LdPepV) (24).

**Figure 2:**
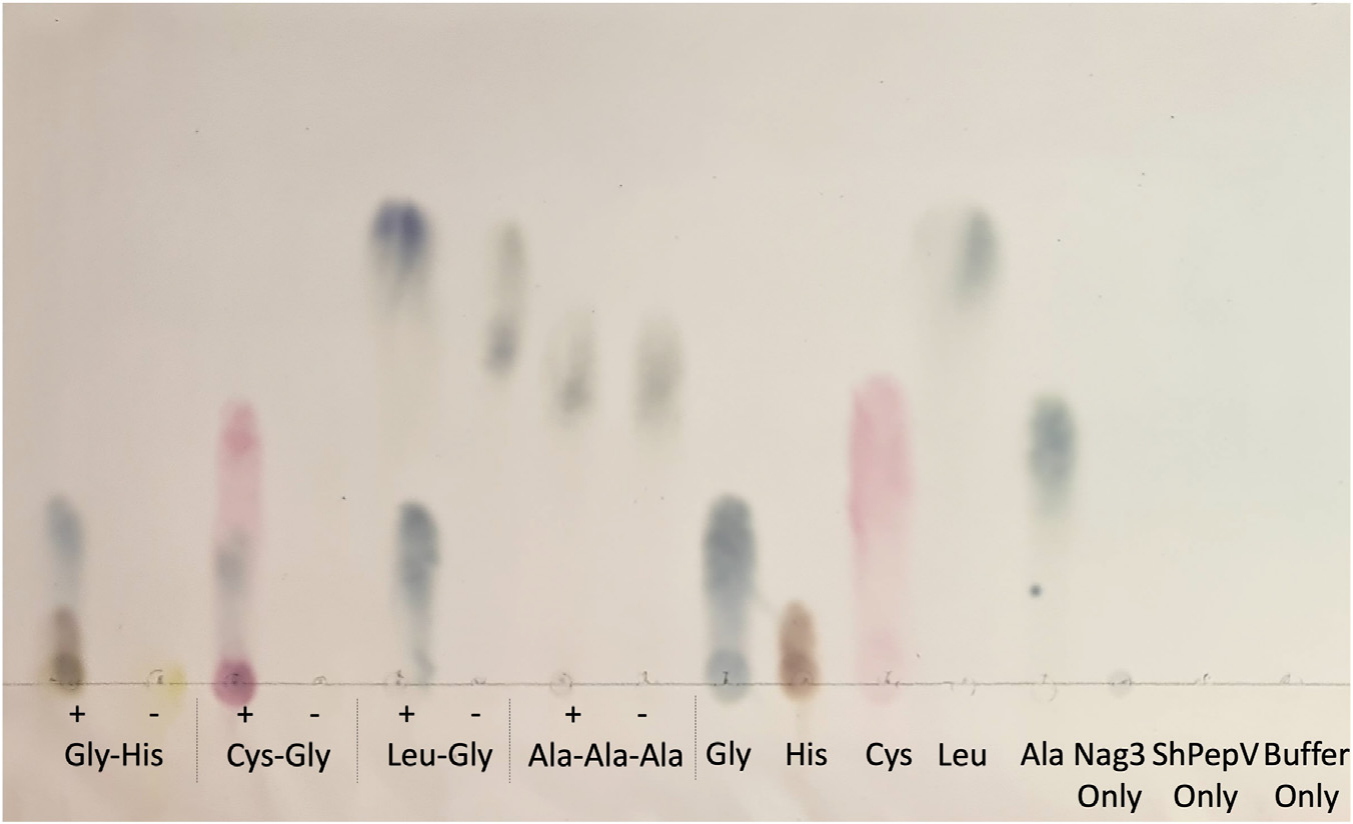
**TLC-based assay of ShPepV activity against a subset of dipeptides tested.** Peptides were incubated with (+) or without (−) PepV. Relevant protein and amino acid controls were included. All substrates and controls were solubilized in dH_2_O. Other TLC plates are shown in Supporting informations S9 and S10.

The substrate specificity of ShPepV was assessed using a range of commercially available dipeptides with varying chemistries and sizes at the P1 and P1′ positions (Table S4). With the exception of D-Ala-D-Ala, which has a very different stereochemistry to the other L-amino acid–containing peptides, ShPepV was capable of cleaving all dipeptides tested (Fig. 2 and Datas S9 and S10). However, it was unable to cleave the tripeptide L-Ala-L-Ala-L-Ala, suggesting preference and selectivity for dipeptide substrates (Fig. 2).

### ShPepV and SaPepV are efficient Cys-Gly-3M3SH dipeptidases

**Figure 3:**
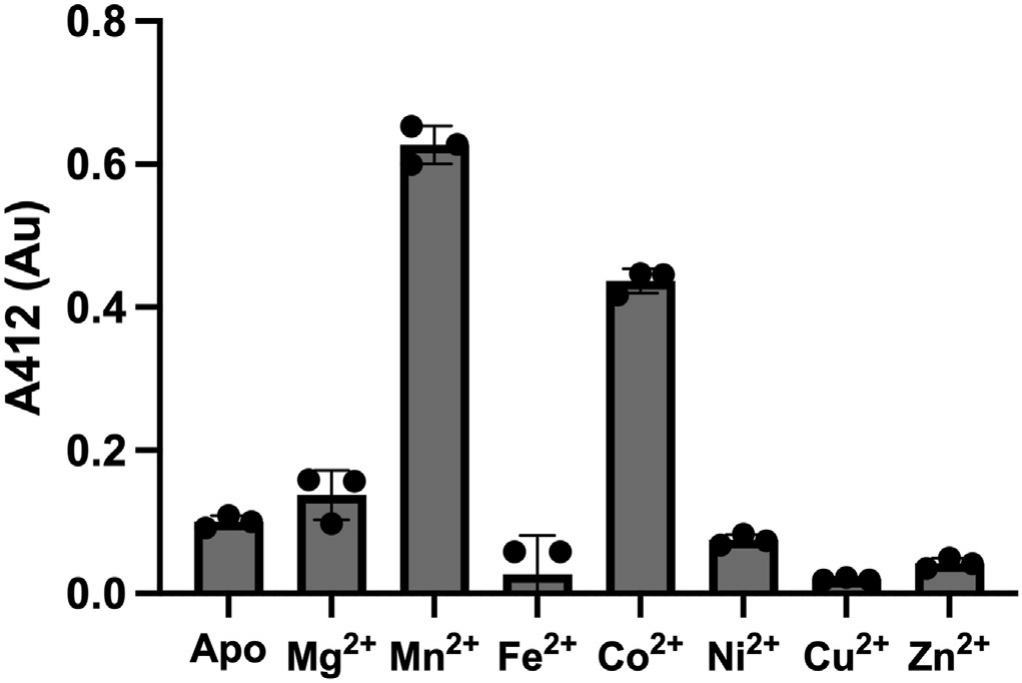
**The dipeptidase activity of ShPepV against Cys-Gly-3M3SH in the presence of various divalent cations reveals a preference for Mn**^**2+**^**as the metal cofactor.** A negative control (Apo) containing no added divalent cations was included. Enzyme activity was determined using the coupled DTNB assay (data in technical triplicates with errors [SD]). DTNB, 5,5′-dithiobis-(2-nitrobenzoic acid); 3M3SH, 3-methyl-3-sulphanylhexan-1-ol.

We next monitored the kinetics of PepV catalysis against malodor substrates. The study of SaPepV by Girish and Gopal (29) revealed that the protein is metal dependent with highest catalytic activity when purified in the presence of Mn^2+^. To determine if ShPepV behaves similarly, we removed copurifying metals from the recombinantly purified protein through dialysis against an EDTA-containing buffer (apo-ShPepV) and added a range of divalent metals to the protein before testing activity against Cys-Gly-3M3SH. We observe striking resemblance to the findings of Girish and Gopal (29) with Mn^2+^-bound ShPepV exhibiting the highest activity with Co^2+^ having the second highest (Fig. 3). In light of this, downstream enzyme assays of ShPepV and SaPepV were carried out with proteins purified in the presence of Mn^2+^ and Tris(2-carboxyethyl)phosphine (TCEP).

Using the coupled DTNB assay, we determined the kinetic parameters of the two staphylococcal PepV proteins with Cys-Gly-3M3SH as the substrate (Table 1). For comparison, we expressed and purified the previously characterized Cys-Gly-3M3SH dipeptidase TpdA (17) from *Corynebacterium glaucum* DSM 30827 (31) as a known positive control (CgTpdA).

**Table 1:** Kinetics of ShPepV, SaPepV, and CgTpdA with Cys-Gly-3M3SH and ShPepV with Cys-Gly

Protein	Substrate	Assay used	*k*_cat_ (s^−1^)	*K*_*m*_ (mM)	*k*_cat_*/K*_*m*_ (s^−1^ mM^−1^)
ShPepV	Cys-Gly-3M3SH	Coupled DTNB	3.41 ± 0.07	1.29 ± 0.04	2.64 ± 0.10
SaPepV	Cys-Gly-3M3SH	Coupled DTNB	2.96 ± 0.14	3.34 ± 0.34	0.89 ± 0.10
CgTpdA	Cys-Gly-3M3SH	Coupled DTNB	1.20 ± 0.07	0.48 ± 0.03	2.50 ± 0.85
ShPepV	Cys-Gly	Cd–ninhydrin	405.40 ± 23.56	2.19 ± 0.22	186.00 ± 21.76

The assay used for kinetic parameter determination is also included. Kinetic values and errors (SD) reported were corrected to 2 s.f.

ShPepV displayed the highest catalytic efficiency (*k*_cat_/*K*_*m*_) of the three enzymes with TpdA exhibiting comparable activity against Cys-Gly-3M3SH (Table 1 and Data S11, *B*–*D*). Interestingly, the catalytic efficiency (*k*_cat_/*K*_*m*_) of SaPepV with Cys-Gly-3M3SH is approximately threefold lower than that of ShPepV. While the *k*_cat_ remains comparable, the weaker *K*_*m*_ values derived for the *S. aureus* homolog seems to be contributing to the lower catalytic efficiency suggesting the enzyme is slightly less capable of accommodating Cys-Gly-3M3SH.

To determine the effects of the large 3M3SH group in the catalytic activity of ShPepV with Cys-Gly-3M3SH, we compared its activity to that against the dipeptide Cys-Gly. To measure this, we used a previously reported Cd–ninhydrin method (32) to detect free amino acids (Table 1 and Data S11*A*). Surprisingly, the *K*_*m*_ was approximately twofold more than with Cys-Gly-3M3SH suggesting a slightly less preferable binding. However, the enzyme turnover number (*k*_cat_) with Cys-Gly was almost 100-fold higher than with Cys-Gly-3M3SH, which resulted in an overall efficiency increase of ∼70-fold. We have demonstrated that ShPepV is capable of cleaving Cys-Gly-3M3SH at similar rates exhibited *in vitro* by the TpdA enzymes from *C. glaucum* DSM 30827 (29) but is far superior with the simpler Cys-Gly dipeptide.

### ShPepV is general dipeptidase that can accommodate Cys-Gly-3M3SH

**Figure 4:**
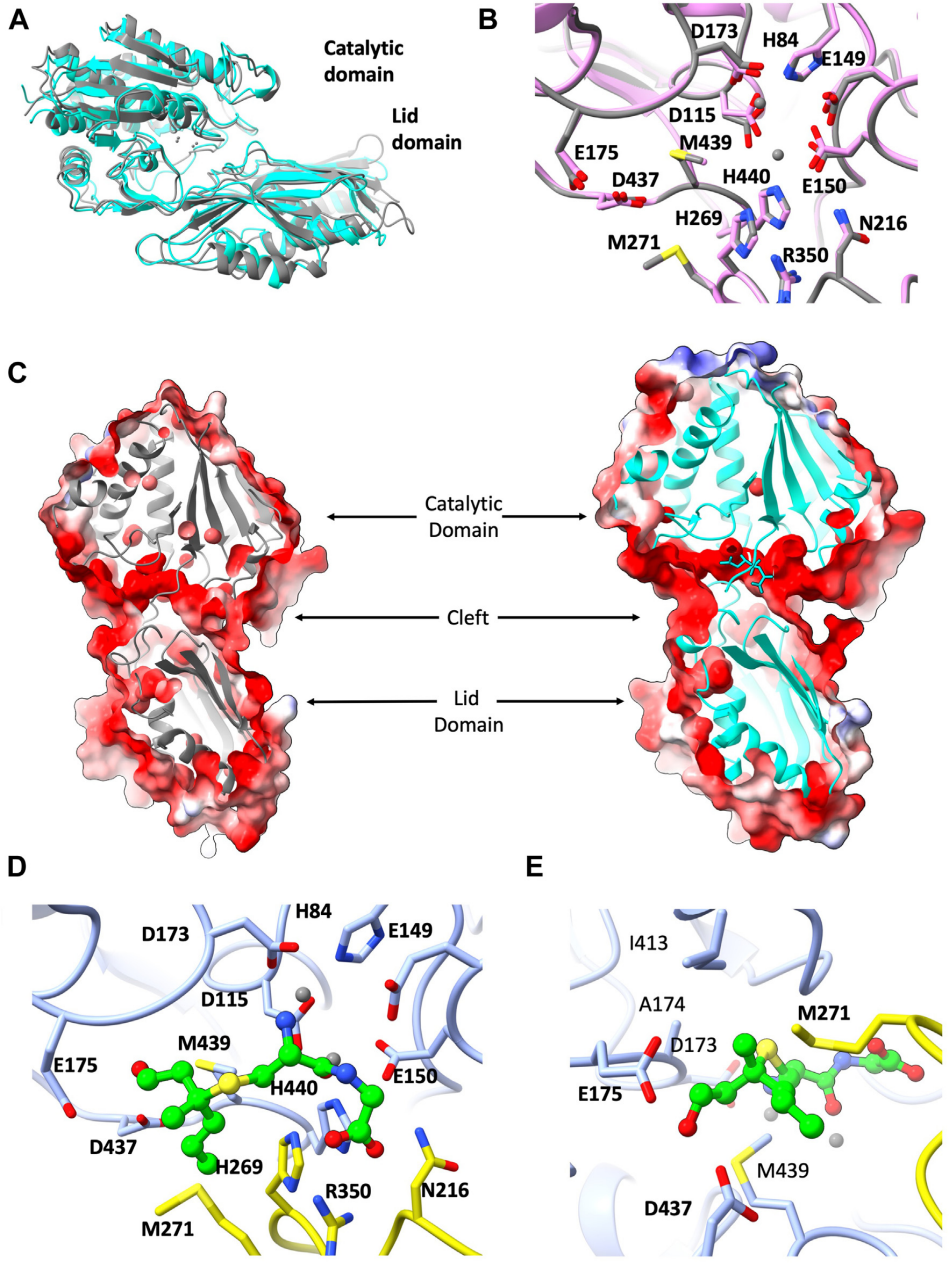
**The structural model of ShPepV possesses a binding pocket capable of accommodating Cys-Gly-3M3SH.***A*, the model of ShPepV (*gray*) suggests the canonical PepV fold with a catalytic and lid domain that is observed in the experimentally derived structure of LdPepV (*cyan*) (Protein Data Bank code: 1LFW, RMSD = 1.66 Å over 447 residues). *B*, the putative binding site of ShPepV (*gray*) shares the same residues as SaPepV (*pink*). *C*, surface representations of ShPepV (*gray ribbon*) and LdPepV (*cyan ribbon*) reveal a common substrate binding cleft. *D*, using a rigid-body refinement approach, Cys-Gly-3M3SH (*ball and stick representations*) was modeled into the binding site of ShPepV using the known positions of the Asp-Ala inhibitor in LdPepV. *E*, a rotated view of the modeled ligand binding site indicating various buried and surface (*bold*) residues that were mutagenized. For (*D* and *E*) the carbon backbone of the catalytic domain of ShPepV is represented in *light blue* and that of the lid domain is in *gold*. Images were generated with ChimeraX 1.8 (54). 3M3SH, 3-methyl-3-sulphanylhexan-1-ol.

The ability of ShPepV to function using Cys-Gly-3M3SH as a substrate suggests that the active site accommodates the bulky (S)-conjugate even though it is unlikely to have evolved primarily for this function. To address this hypothesis, we exploited the high sequence identity between ShPepV and SaPepV (79.4% identity and 90.2% similarity) and used the existing structure of the latter (Protein Data Bank [PDB] code: 3KI9) (29) as a template for homology modeling of ShPepV. The model of ShPepV was generated with high confidence. The Global Model Quality Estimation score was determined to be 0.91, which suggests high confidence of the generated tertiary structure. The QMEANDisCo Global value was calculated to be 0.86 ± 0.05, which suggests high per-residue quality for the model against the template. The overall structure of ShPepV conforms to the canonical PepV fold consisting of a catalytic domain and a “lid” domain enclosing a putative metal containing active site (Fig. 4*A*). As expected, both staphylococcal PepVs share identical residues in their putative active sites, and hence, the two metal cofactors expected in the binding site (Fig. 4*B*). However, despite the high sequence identities of ShPepV and SaPepV, the latter exhibited weaker dipeptidase activity with Cys-Gly-3M3SH (Table 1, column *k*_cat_/*K*_*m*_).

Our previous structural studies demonstrated the importance of largely hydrophobic cavities in the transporter DtpT (15) and the C-S β-lyase PatB (16) for the selective accommodation of the largely hydrophobic 3M3SH moiety. The model of ShPepV reveals an interdomain cleft, which passes through the protein and is likely to be the peptide binding site (Fig. 4*C*). A similar cleft was observed in the cross-section of the experimentally derived structure of *L. delbrueckii* PepV (LdPepV; PDB code: 1LFW), containing the inhibitor Asp-Ala phosphinic acid (24). Using this structure and the position of the dipeptide mimic, we performed a manual rigid body refinement to suggest a fit for Cys-Gly-3M3SH into the model of ShPepV. We observed highly conserved residues with roles in the binding of the dipeptide backbone of both structures (Data S12). The proposed binding cleft accommodates Cys-Gly-3M3SH with the major protein interactions expected to be with the dipeptide backbone (Fig. 4*D*). The 3M3SH moiety of the fitted ligand extends out of the binding site into a large cavity, which can accommodate the bulky hydrophobic group within the allowed molecular restraints.

### Mutagenesis of the ShPepV binding pocket reveals key Cys-Gly-3M3SH accommodating residues

When fitting the hydrophobic part of 3M3SH, which includes a quaternary carbon, there were very few possible positions it could adopt in the binding cavity. The 3M3SH moiety seem to fit best in a pocket circumscribed by residues A174, I413, M439, D437, E175, and M271. This allowed us to identify residues that might modulate the quality of the fit of the ligand into the binding pocket (Fig. 4*E*). To test this, we recombinantly expressed ShPepV with these residues replaced by either smaller or larger residues to increase or decrease the size of the cavity, respectively, at its base or the surface. Consistent with the WT protein, these mutants were also purified in the presence of Mn^2+^ and TCEP.

**Figure 5:**
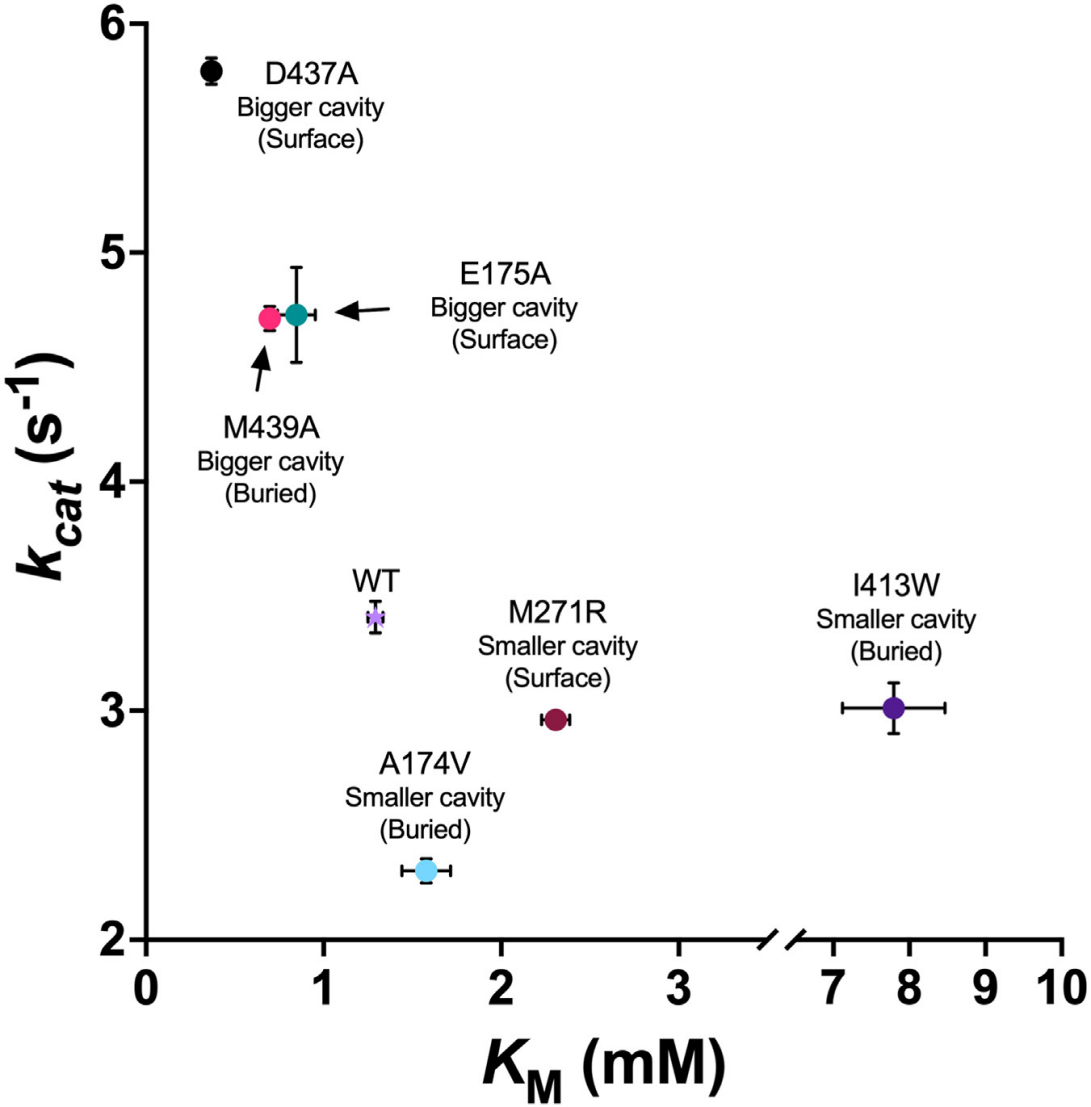
**Plot of *k***_**cat**_**against *K***_***m***_**of ShPepV mutants with the WT protein.** Mutants that constrict the binding site (A174V, M271R, and I413W) result in varying reductions in affinity for Cys-Gly-3M3SH (higher *K*_*m*_). Conversely, ShPepV mutants resulting in a bigger cavity (D437A, M439A, and E175A) have improved affinity values for Cys-Gly-3M3SH. 3M3SH, 3-methyl-3-sulphanylhexan-1-ol.

Enlarging buried residues in the cavity through the substitutions A174V and I413W should severely restrict the volume around the quaternary carbon. The mutants retained 55% and 14.6% activity, respectively, when compared with the WT protein (Table 2). The *K*_*m*_ derived for the I413W mutant was almost eightfold worse than the WT protein suggesting that ligand accommodation is significantly hampered by the much larger tryptophan residue (Fig. 5, Table 2, and Data S13*A*). A complete loss of activity against Cys-Gly-3M3SH is seen with a mutant of a metal coordinating residue D173A (Table 2). This was expected as the metal cofactor is thought to activate water for nucleophilic attack and/or polarize the peptide carbonyl for this attack.

**Table 2:** Kinetics of ShPepV mutants with Cys-Gly-3M3SH determined using the coupled DTNB assay

Protein	Purpose	*k*_cat_ (s^−1^)	*K*_*m*_ (mM)	*k*_cat_*/K*_*m*_ (s^−1^ mM^−1^)	(*k*_cat_*/K*_*m*_)_mut_/(*k*_cat_*/K*_*m*_)_WT_	ΔΔG^≠^ (kJ.mol^−1^)
WT	Native	3.41 ± 0.07	1.29 ± 0.04	2.64 ± 0.10	—	—
D173A	Metal coordinating	ND	ND	ND	—	—
A174V	Smaller cavity (buried)	2.30 ± 0.05	1.58 ± 0.14	1.46 ± 0.13	0.55	−1.54
I413W	Smaller cavity (buried)	3.01 ± 0.11	7.79 ± 0.67	0.39 ± 0.04	0.146	−4.96
M439A	Bigger cavity (buried)	4.71 ± 0.05	0.70 ± 0.01	6.77 ± 0.13	2.56	2.42
D437A	Bigger cavity (surface)	5.79 ± 0.06	0.37 ± 0.03	15.7 ± 1.27	5.96	4.60
E175A	Bigger cavity (surface)	4.73 ± 0.21	0.85 ± 0.11	5.59 ± 0.75	2.11	1.93
M271R	Smaller cavity (surface)	2.96 ± 0.03	2.31 ± 0.08	1.28 ± 0.05	0.486	−1.86

ND, not detectable.

The fold change in catalytic efficiencies ((*k*_cat_*/K*_M_)_mut_/(*k*_cat_*/K*_M_)_WT_) and differences in transition state binding energies (ΔΔG^≠^) of the mutants against that of the WT protein were also calculated.

Conversely, the M439A mutant, which is expected to result in a larger binding site and retention of activity, leads to a marginal increase in *k*_cat_ and a ∼2-fold decrease in the *K*_*m*_ providing a twofold improvement in *k*_cat_/*K*_*m*_ when compared with the WT protein (Fig. 5, Table 2, and Data S13*B*). Continuing this logic, we also reduced the size of other residues that our model predicted might restrict the active site, specifically E175 and D437. Altering the first two of these residues to an alanine slightly improved the *k*_cat_ of the resulting enzymes with *K*_*m*_ increasing by ∼1.5-fold and ∼3.5-fold, respectively (Fig. 5, Table 2, and Data S13, *C* and *D*). Similarly, the residue enlarging M271R mutation resulted in a ∼2-fold increase in *K*_*m*_ while retaining a similar turnover number (*k*_cat_). Together, these data suggest our structural model for the enzyme has some validity and by being able to actually increase the catalytic efficiency of ShPepV against Cys-Gly-3M3SH, that the enzyme clearly has not evolved to recognize this as its primary substrate.

We generally observe similar patterns of fold changes in the catalytic efficiencies of ShPepV mutants against the WT protein ((*k*_cat_*/K*_*m*_)_mut_/(*k*_cat_*/K*_*m*_)_WT_) with the dipeptide Cys-Gly (Table S5 and Data S14). The activity is unchanged in the A174V mutant when compared to the WT protein, which suggests this mutant is less important in the accommodation of the thiol group of the cysteine moiety of Cys-Gly than in the accommodation of the quaternary carbon conjugated sulfur of Cys-Gly-3M3SH. However, the I413W mutant only retained 0.0025% of the WT protein activity with the *k*_cat_ largely contributing to the decrease. In comparison, the same mutant led to only an approximately sevenfold decrease in enzymatic efficiency (14.6% activity retention) when compared with the WT protein with Cys-Gly-3M3SH (Table 2). Clearly but unexpectedly, this mutation has drastically affected the reaction involving the simpler dipeptide. Overall, our data suggest that the binding site could not only be improved for the complex malodor precursor Cys-Gly-3M3SH but also the simpler dipeptide Cys-Gly.

## Discussion

**Figure 6:**
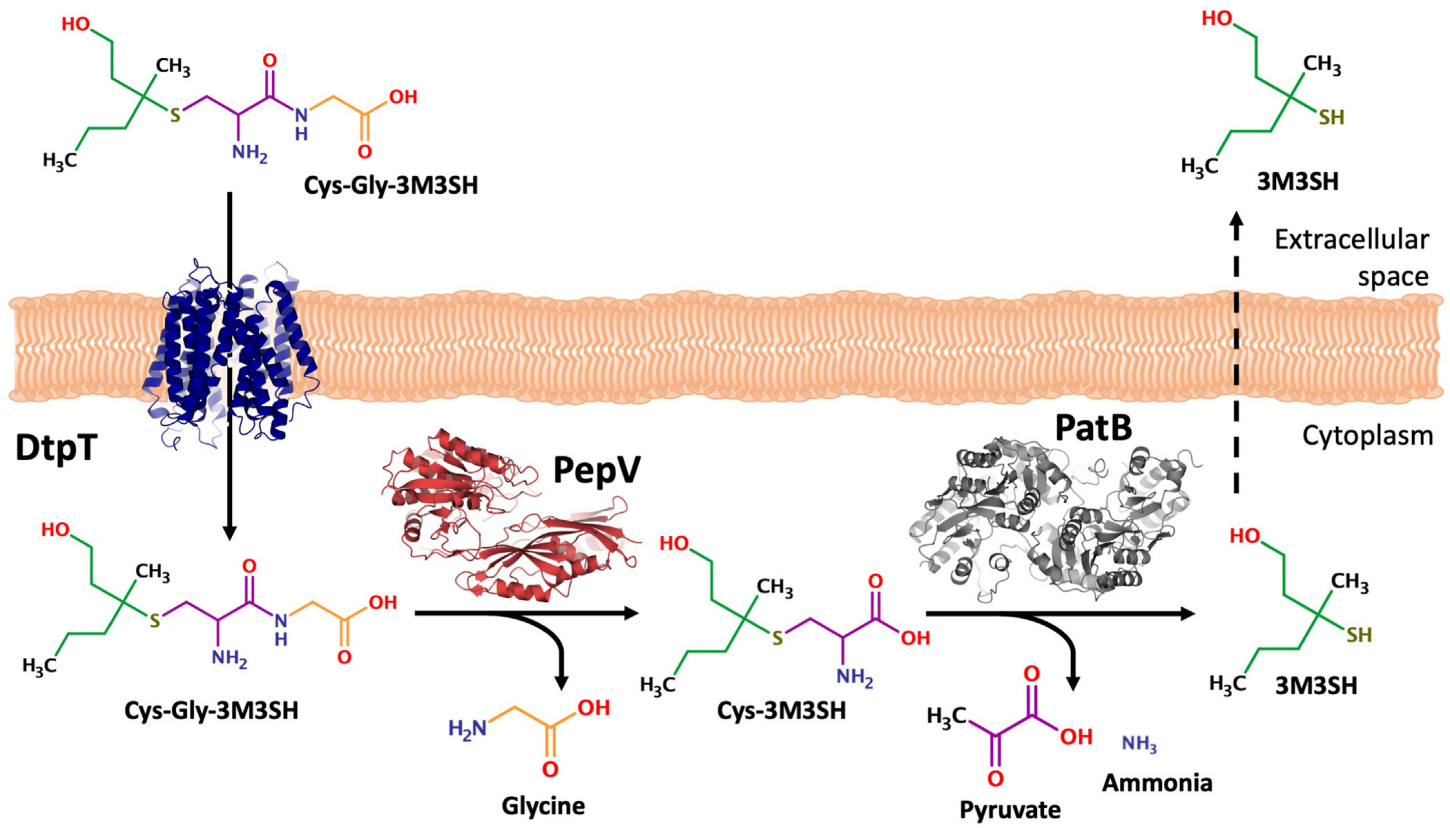
**Uptake and enzymatic processing of Cys-Gly-3M3SH in *Staphylococcus hominis*.** The odorless human-derived metabolite Cys-Gly-3M3SH is taken into the bacterial cell by the proton-dependent oligopeptide transporter (POT) family transporter DtpT. The general dipeptidase PepV cleaves the glycine off before the C-S β-lyase activity of PatB releases the pungent molecule 3M3SH, which escapes the cell through an unknown mechanism. C-S β-lyase, cysteine-S-conjugate β-lyase; 3M3SH, 3-methyl-3-sulphanylhexan-1-ol.

Since the discovery of the important role staphylococci play in the generation of thioalcohol-based human malodor derived from the axillary metabolite Cys-Gly-3M3SH [5,19], a number of studies have helped identify and characterize proteins involved in the biotransformation of this molecule (15, 16, 17, 18). The proton-dependent oligopeptide transporter family transporter DtpT is involved in the cellular uptake of the odorless Cys-Gly-3M3SH into the cytoplasm (15), where a hitherto unknown dipeptidase and the C-S β-lyase PatB (16) cleave this molecule in tandem to produce the pungent 3M3SH (Fig. 6). Here, we identified the Cys-Gly-3M3SH active dipeptidase ShPepV empirically through chromatography techniques with the aid of a coupled DTNB assay. Homologs of this protein were previously reported in the literature to be nonspecific L-amino acid dipeptidases with no apparent selectivity for or against dipeptides with large side chains on the N terminus (24, 25, 29). Assays of the purified recombinant enzyme confirmed the dipeptidase activity of ShPepV with Cys-Gly-3M3SH. Using structural modeling, we identified a clear interdomain substrate binding cleft and suggested a cavity capable of accommodating the bulky 3M3SH group. This possible location was supported by mutagenic studies on the putative pocket, which when enlarged, allowed for a more conducive fit as seen by an increase in apparent affinity (*K*_*m*_). In contrast, when the pocket size is reduced, there is reduction of activity (Table 2). In addition, we also unexpectedly noted increases in *k*_cat_ of mutants that enlarges the binding site. These residues could play a role in the interdomain interactions, which in turn controls the enzyme turnover. Clearly, our mutations created a more favorable environment for the dipeptidase activity with Cys-Gly-3M3SH by having improved transition state binding energies when compared to the WT protein (Table 2, column ΔΔG^≠^). This suggest that PepV from *S. hominis* could have easily evolved to improve Cys-Gly-3M3SH turnover, but this was not observed. We suggest that the dipeptidase step may not be the rate-limiting step of the reaction, and hence, does not need to be improved. Alternatively, the general ability for *S. hominis* to biotransform Cys-Gly-3M3SH might not be essential for cell survival in the axillary environment.

Despite the apparent ability for accommodation of an extended dipeptide like Cys-Gly-3M3SH, ShPepV was not able to cleave L-Ala-L-Ala-L-Ala. Vongerichten *et al.* (25) also observed this with LdPepV and found only a handful of other tripeptides that are able to be hydrolyzed by this dipeptide preferring enzyme. In our model, we observe the N terminus of the dipeptide backbone facing D173 and H84, and the same was observed with the inhibitor-bound structure of LdPepV (Fig. 4*D* and Data S12). Together, this suggest that PepV is a true dipeptidase.

The staphylococcal PepV enzymes are highly conserved with likely roles in metabolizing exogenous and endogenous peptides and potentially in antibiotic resistance (29, 30). We have identified PepVs in a wide range of skin-associated staphylococci including the commonly found opportunistic pathogens *Staphylococcus epidermidis* and *S. aureus*. Alongside ShPepV, we followed on from previous characterization studies of the *S. aureus* homolog SaPepV (29, 30) to characterize its potential Cys-Gly-3M3SH dipeptidase ability. The residues that form the likely active site of both ShPepV and SaPepV were found to be identical in both the primary sequence and the tertiary structure (Fig. 4*B*). Consistent with our observations with ShPepV, we found SaPepV to be enzymatically active against Cys-Gly-3M3SH.

Clearly, proteins possessing functional capabilities in the biotransformation of Cys-Gly-3M3SH can exist in multiple species and strains that are found in the underarm, potentially giving them access to useful nutrients for growth using host secretions. However, as of yet, it is still not clear as to the selective advantage, if any, of metabolizing these malodor precursors. Malodor precursors like Cys-Gly-3M3SH and Gln-3M2H are released from the apocrine gland *via* the protein ABCC11 (33). Multiple genetic studies have identified SNPs at amino acid position 180 of ABCC11, which limits its ability to export these malodor precursors in people in some Asian communities (33, 34, 35). These individuals with lower axillary odor seemed to have a change in the distribution of genera within the axillary microbiome, shifting slightly from staphylococci and anaerococci to corynebacteria (36). This shift in population could be attributed to a change in the metabolites available because of the absence of a functioning ABCC11. Most skin-resident staphylococci would stand to benefit from the products of the biotransformation of Cys-Gly-3M3SH, as resultant glycine from the PepV reaction can be used directly for protein synthesis (37) but also forms the major part of the crosslink between the glycan chains of the staphylococcal peptidoglycan (38, 39).

To date, we are unaware of how the expression of *dtpT*, *pepV*, and *patB* is regulated. The genes are not clustered on the genome of *S. hominis* suggesting that the functions of the gene products are not solely for this pathway. Unlike the PatB enzyme, neither the transporter DtpT nor the peptidase PepV seem to be uniquely adapted for Cys-Gly-3M3SH biotransformation, further supporting the notion that this pathway piggybacks on other general cellular functions. In addition, while we purified PepV from an activity assay and were able to clearly measure its activity on Cys-Gly-3M3SH, we cannot say if this is the only peptidase in the cell that is active on this substrate. We are not able to truly replicate the conditions in the underarm *in vitro* nor is it feasible for us to perform genetic manipulations on this genetically intractable species of *Staphylococcus*. Hence, it is possible that other related enzymes also make some contribution to this process *in vivo*. Nonetheless, the ability to transport short peptides and to then cleave them into free amino acids in the cytoplasm is likely an important core nutritional function for skin-dwelling staphylococci, and indeed, there is evidence that the peptide transporters Opp and DtpT are required for host colonization (37, 40, 41, 42). The function of peptidases must also be essential to use these transported peptides, but the multiplicity of these systems in the cell means that it is unlikely that removal of a single system would result in a strong cellular phenotype. However, there is some evidence for specialization of peptidase function as PepV itself has been linked to β-lactam resistance (30), presumably by cleaving β-lactams intracellularly, while other more specific aminopeptidases are known to be important for *S. aureus* virulence (43).

In conclusion, this study provides evidence for the missing mechanistic step in the full pathway for the production of thioalcohol-based malodor in *S. hominis*. As DtpT and PepV are found widely in staphylococci, it supports our previous finding that the occurrence of *patB*, encoding the cysteine-(S) β-lyase, correlates very strongly with malodor production in staphylococci (16).

## Experimental procedures

### Bacterial strains and plasmids

Bacterial strains and plasmids used in this study are as listed in Tables S1 and S2 (44, 45, 46, 47). In this study, all growth of *Escherichia coli* was performed using LB media, whereas staphylococci and corynebacteria were grown in tryptic soy broth media.

### Isolation and identification of ShPepV

A 1 l overnight culture of *S. hominis* B10 was grown at 37 °C, with shaking in a baffled flask. The cells were then pelleted and resuspended in 20 mM Tris–HCl, pH 8, supplemented with 5 μg Lysostaphin (Merck). The suspension was incubated for an hour at 37 °C before being sonicated to lyse the cells further. The lysate was clarified by centrifugation, and the supernatant was loaded onto a 4.7 ml HiScreen CaptoQ AEX column (Cytiva). Proteins were then fractionated using a 0 to 1 M NaCl linear gradient in 20 mM Tris–HCl (pH 8.0). Each fraction from this and all subsequent purification steps was analyzed for activity using a coupled DTNB assay as described later (Data S1). Fractions with the highest activity were pooled and buffer exchanged into 50 mM potassium phosphate (Kpi) (pH 7.8) and 1 M (NH_4_)_2_SO_4_.

The proteins were then resolved by hydrophobic interaction chromatography on a HiScreen Phenyl HP hydrophobic interactions chromatography column (Cytiva). After washing, proteins were eluted using a descending (1–0 M) (NH_4_)_2_SO_4_ linear gradient in 50 mM Kpi (pH 7.8) (Data S2). Fractions with the highest activity were buffer exchanged into 20 mM Bis–Tris (pH 7.0).

The proteins were further fractionated by a gradient elution into 20 mM Bis–Tris (pH 7), 1 M NaCl using a HiScreen CaptoDEAE AEX column (Cytiva). The coupled DTNB assay was repeated as previously but incubated for an hour (Data S3). Fractions with the highest activity were finally loaded onto a HiPrep S200 Sephacryl size-exclusion chromatography column (Cytiva) (Data S4).

The fraction with the highest activity, the two fractions preceding and the two fractions succeeding were concentrated, activity confirmed by the coupled DTNB assay as aforementioned (Data S6), and the remaining proteins were loaded onto a 12% SDS-PAGE gel. The resulting gel was Coomassie stained, and image was captured on a gel-doc system. The protein band consistent with the activity seen in the fraction was excised, eluted from the gel, and digested with trypsin. The resulting peptide fragments were analyzed by MALDI–MS/MS and searched against all UniProt entries within the species *S. hominis*. Three distinct and one modified peptide fragments matched to the dipeptidase PepV (accession number: A0A657M1L9).

### Cloning, expression, and purification of ShPepV, SaPepV, and CgTpdA

Genomic DNA of *S. hominis* B10, *S. aureus* USA300 JE2, and *C. glaucum* DSM30827 was isolated using the Dneasy Blood and Tissue Kit (Qiagen) with either lysostaphin or lysozyme used to digest the cell walls. The *pepV* genes from the staphylococci and *tpdA/dapE3* from the corynebacteria were amplified by PCR (Table S3) and cloned into pBADcLIC using the ClonExpress II One Step Cloning Kit (Vazyme). This recombinant expression system introduces a C-terminal tag (-ENLYFQGHHHHHHHHHH). For protein production, these plasmids were introduced into *E. coli* MC1061. Overnight cultures were used for inoculation into fresh 1 l LB supplemented with 100 μg mL^−1^ ampicillin. Cultures were grown to an absorbance of ∼0.5 at 600 nm at 37 °C before the induction of recombinant protein production with 0.01% l-arabinose. The culture was allowed to grow for a further 5 h at 30 °C. Cells were then harvested by centrifugation, and the resulting pellet was resuspended in purification buffer A (50 mM Kpi [pH 7.8], 200 mM NaCl, 20% glycerol, and 40 mM imidazole). The cells were then sonicated at 250 W with a 30% duty cycle for 10 min. Clarification of the lysate was then performed at 27,000*g* for 30 min. Soluble protein fractions were then passed through a pre-equilibrated HisTrap FF crude column (Cytiva). The column was washed with purification buffer A over 10 column volumes, and the bound proteins were eluted with purification buffer B (50 mM Kpi [pH 7.8], 200 mM NaCl, 20% glycerol, and 500 mM imidazole). Prior to downstream experiments, the proteins were buffer exchanged into 20 mM Tris–HCl (pH 8) and 50 mM NaCl. The Mn^2+^-bound protein used for the kinetics experiments was prepared as described previously but with 1 mM MnCl_2_ and 3 mM TCEP added to all purification buffers.

### Mutagenesis of ShPepV

Mutants of the recombinantly expressed ShPepV were generated by inverse PCR using the mutagenic primer pairs mentioned in Table S3 with pBcL::Sh*pepV* as the template. PCR products were circularized by blunt end ligation using 100 ng of DNA, 1 μl T4 DNA ligase (NEB), 1 μl T4 polynucleotide kinase (NEB) in T4 DNA ligase buffer (NEB), and incubated overnight at 16 °C. The ligated products were transformed into *E. coli* XL1-Blue and then sequence verified. Verified plasmids were then transformed into *E. coli* MC1061 for protein production and purification in the presence of Mn^2+^ as mentioned in the previous section.

### Coupled Cys-Gly-3M3SH DTNB assay

A coupled DTNB assay was used to detect peptidase activity against Cys-Gly-3M3SH (Data S5). Reaction mixes contained 1 mM Cys-Gly-3M3SH (Concept Life Sciences), 1 mM purified ShPatB, 0.4 mM DTNB, and 0.1 M Tris–HCl (pH 80). Assays performed for the identification of the peptidase included the elution fractions at 25% (v/v). *In vitro* characterization of ShPepV, its homologs, and its mutants were performed with the same assay mixture containing 1 mM purified enzyme. Reaction mixtures were incubated at 37 °C for 30 min, and the absorbance at 412 nm was measured using an Epoch 2 Spectrophotometer with a minimum detectable change of 0.0001 absorbance (BioTek/Agilent).

The steady-state kinetics of the PepV dipeptidase activity on Cys-Gly-3M3SH was monitored using the coupled DTNB assay with varying concentrations of Cys-Gly-3M3SH in a 96-well plate. The absorbance at 412 nm values was measured every 15 s on a microplate spectrophotometer to determine the initial rate of substrate turnover for each concentration of ligand. The measured absorbance at 412 nm values corresponds to TNB, which is generated in a 1:1 ratio to every 3M3SH generated. Michaelis–Menten plots were generated to derive the *k*_cat_ and *K*_*m*_ using Prism 10 (GraphPad). The catalytic efficiencies were then derived by calculating *k*_cat_ upon *K*_*m*_.

### Dipeptide activity assayed by TLC

About 100 mM stocks of peptides and amino acids (Table S4) were prepared in dH_2_O with the exception of l-tyrosine, which was prepared in 1 M HCl. About 20 mM ShPepV was incubated with 20 mM peptides in 20 mM Tris–HCl (pH 8) and 50 mM NaCl for 30 min at 37 °C. Nag3, an M20 peptidase family aminohydrolase from *Anaerococcus nagyae* (accession: WP_117521798), was used as a negative control enzyme. About 3 μl of each reaction mixture and equivalent amounts of the controls were dropped onto glass-backed silica TLC plates. A 90:10 mixture of ethanol:acetic acid was used as the mobile phase in a pre-equilibrated chamber. Following chromatography, the plates were allowed to dry, and a 1% ninhydrin solution (Merck) was sprayed over the plates. The plates were allowed to dry again before color development using a heat gun.

### Metal cofactor test

The Apo-ShPepV used in the metal cofactor test was prepared by dialyzing the purified protein with 10 mM EDTA on ice for 5 h followed by a dialysis over 18 h into a buffer containing 20 mM Tris–HCl (pH 8), 50 mM and 10 mM EDTA. The resulting protein was then buffer exchanged into 20 mM Tris–HCl (pH 8) and 50 mM NaCl. For the assay, 1 mM Apo-ShPepV was added into a buffer containing 20 mM Tris–HCl (pH 8), 50 mM NaCl, 1 mM Cys-Gly-3M3SH, 1 mM purified ShPatB, and 1 mM of either of the following divalent cation salts (MgCl_2_, MnCl_2_, FeCl_2_, CoCl_2_, NiCl_2_, CuSO_4_, and ZnCl_2_) and incubated at 37 °C for 30 min. The Apo-ShPepV without any added divalent cation salts was added as a negative control.

### Kinetics of ShPepV with Cys-Gly

About 1 mM ShPepV was mixed with a range of concentrations of Cys-Gly between 0.125 mM and 2 mM in 50 mM Kpi and 200 mM NaCl in a 96-well microtiter plate. Reactions were incubated at 37 °C for 10 min. Samples were taken at 0, 5, and 10 min, and the reactions were quenched by adding 10 mM of EDTA. About 20 μl of the samples were mixed with 30 μl of 2% ninhydrin and 50 μl H_2_O. The color was developed by incubating the samples at 80°C for 10 min. The absorbance at 570 nm was measured on a microplate spectrophotometer. A standard curve was generated by mixing equimolar concentrations of l-cysteine and glycine between 0.125 mM and 2 mM, incubating the mixture for 10 min at 37 °C and developed with ninhydrin before measurement at 570 nm as aforementioned. The absorbance at 570 nm values measured from the dipeptidase reaction was then compared with those of the standard curve to derive the concentration of Cys-Gly that has been cleaved. A Michaelis–Menten plot was generated to derive the *k*_cat_ and *K*_*m*_ using Prism 9 (GraphPad). The catalytic efficiency was then derived by calculating *k*_cat_ upon *K*_*m*_. The fold changes in *k*_cat_/*K*_*m*_ and the changes in transition state energies (ΔΔG^≠^) of ShPepV mutants against the WT were calculated (48).

### Homology modeling of ShPepV and rigid-body refinement modeling of Cys-Gly-3M3SH

A model of ShPepV was generated on SWISS-MODEL (49, 50, 51) using the known structure of SaPepV (PDB code: 3KI9) (29) as the template because of its high sequence identity at 80%. The Global Model Quality Estimation score was determined to be 0.91, which suggests high confidence of the generated tertiary structure. The QMEANDisCo Global value was calculated to be 0.86 ± 0.05, which suggests high per-residue quality for the model against the template. The electrostatic surfaces were calculated in ChimeraX using Columbic values (ShPepV: minimum = 24.17, mean = 7.01, and maximum = 8.50; LdPepV: minimum = 31.52, mean = 7.25, and maximum = 7.72).

To suggest the likely binding cavity of ShPepV for Cys-Gly-3M3SH, the structure of *L. delbrueckii* PepV bound to an Asp-Ala phosphinic acid inhibitor (PDB code: 1LFW) (24) was aligned with the model of ShPepV. Cys-Gly-3M3SH was fitted into the position of the inhibitor by manual rigid-body modeling in the highly conserved binding sites starting with the carboxy terminus of the peptide, followed by the peptide bond, then the terminal amino group. This positioned the 3M3SH moiety into an adjacent cavity, and a suggested fit of this bulky group was modeled, avoiding clashes with neighboring residues. The ligand was fitted into the binding pocket using Coot (52) within the molecular restraints defined by the program AceDRG (53).

## Data availability

All the data that support the finding in this study are reported either within the article or in the supporting information.

## Supporting information

This article contains supporting information (44, 45, 46, 47).

## Conflict of interest

The authors declare that B. M. is a current employee of Unilever, whereas A. G. J. was an employee of Unilever when this study was carried out. All other authors declare that they have no conflicts of interest with the contents of this article.
