# Supplementary material for: Identification of a staphylococcal dipeptidase involved in the production of human body odor
Source: J Biol Chem. 2024 Oct 24;300(12):107928. doi: 10.1016/j.jbc.2024.107928 (PMC11742315; doi:10.1016/j.jbc.2024.107928)

**Supporting Information**


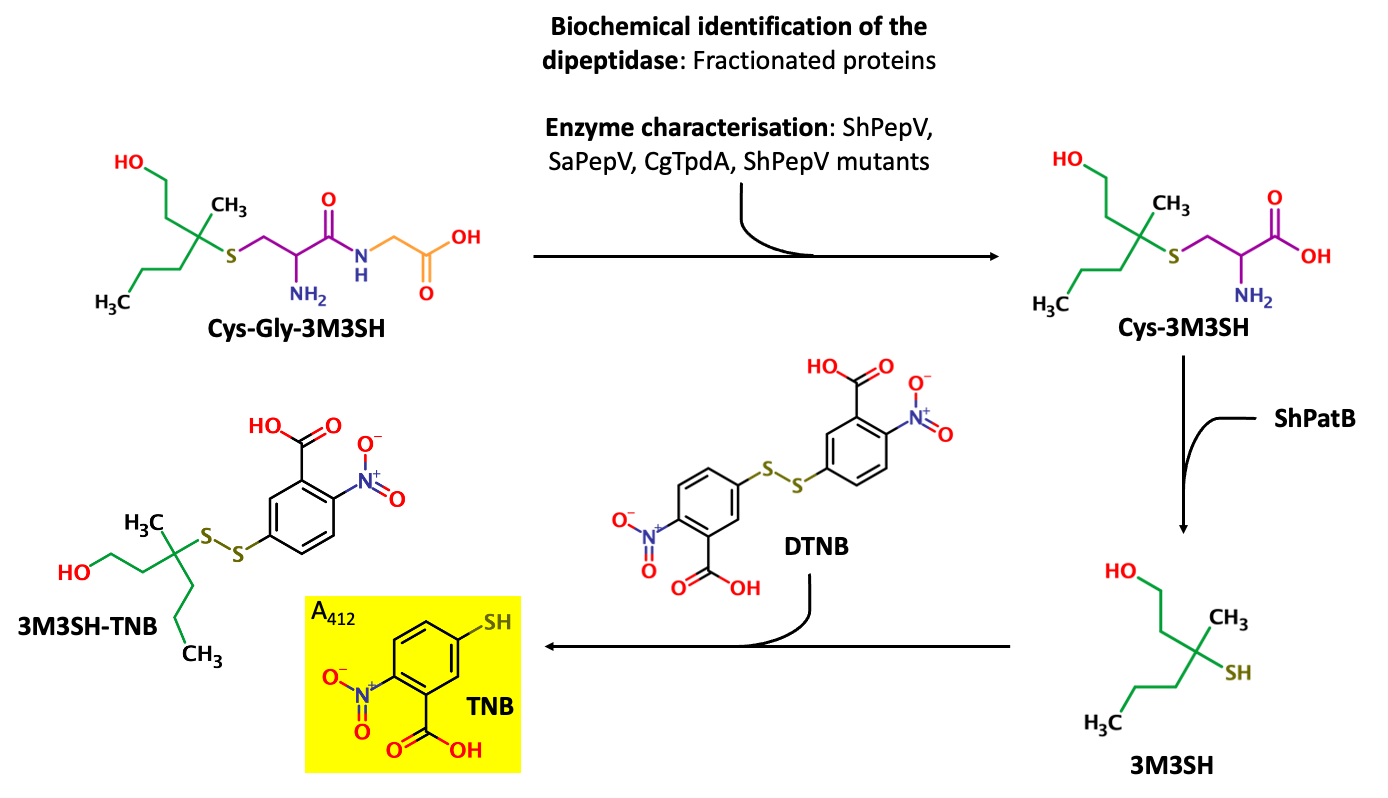


**Supplementary Data S1**: The coupled Cys-Gly-3M3SH DTNB assay. To test the dipeptidase activity, Cys-Gly-3M3SH was either incubated with the fractionated proteins from the various steps of the biochemical identification of the dipeptidase or subsequent recombinantly expressed proteins. ShPatB was added to process any resultant Cys-3M3SH to form 3M3SH which in turn interacts with labelling molecule 5,5′-dithiobis-(2-nitrobenzoic acid) (DTNB). This results in the formation of TNB which absorbs at 412 nm.


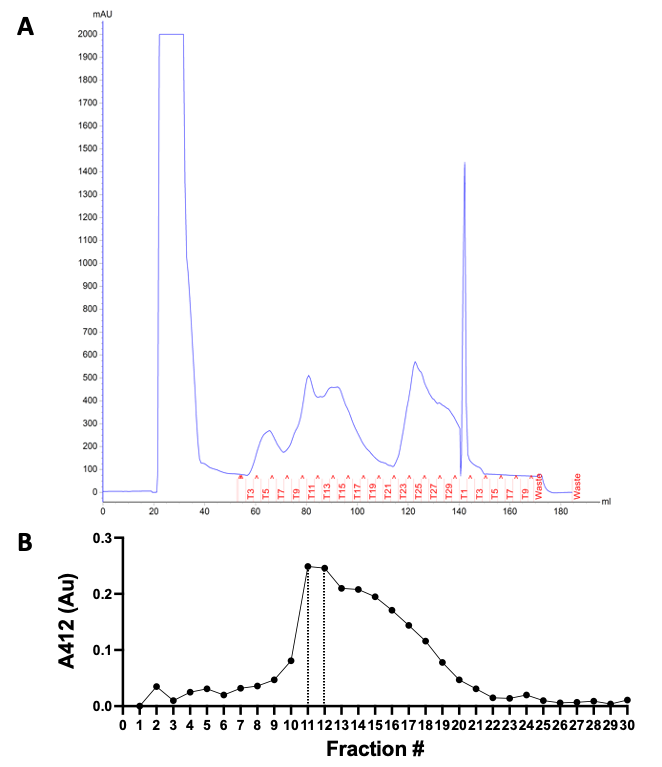


**Supplementary Data S2**. (A) Absorbance at 280 nm measured during anion exchange chromatography with the Capto Q resin (Cytiva). Elution fractions are labelled in red on the X-axis. (B) Elution fractions were tested for Cys-Gly-3M3SH peptidase activity using a coupled DTNB assay. Activity was indirectly measured through the spectrophotometric detection of TNB at 412 nm. Fractions 11 and 12 (black dotted lines) proceeded to the next purification step.


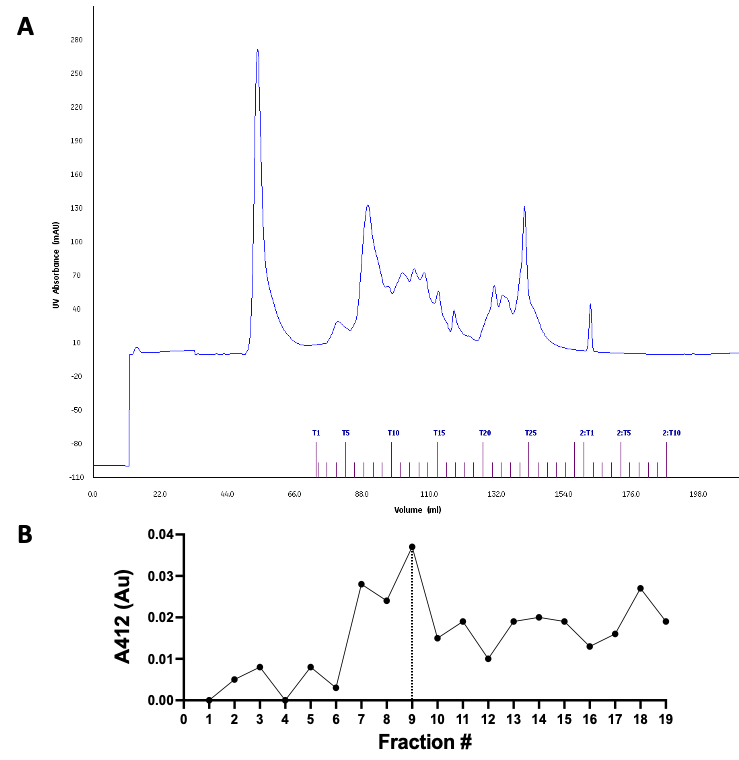


**Supplementary Data S3**. (A) Absorbance at 280 nm measured during hydrophobic interactions chromatography with the Phenyl HP resin (Cytiva). Elution fractions are labelled in red on the X-axis. (B) Elution fractions were tested for Cys-Gly-3M3SH peptidase activity using a coupled DTNB assay. Activity was indirectly measured through the spectrophotometric detection of TNB at 412 nm. Fraction 9 (black dotted line) proceeded to the next purification step.


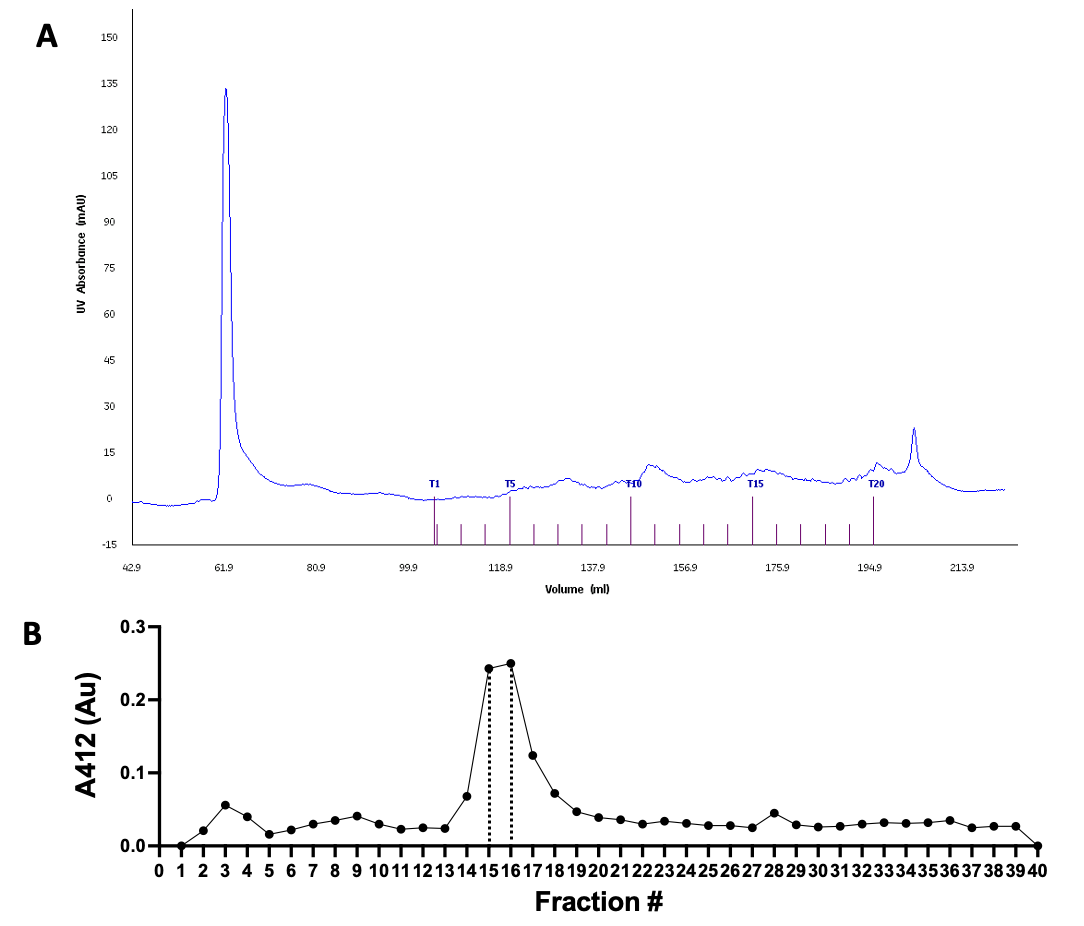


**Supplementary Data S4**. (A) Absorbance at 280 nm measured during anion exchange chromatography with the Capto DEAE resin (Cytiva). Elution fractions are labelled in red on the X-axis. (B) Elution fractions were tested for Cys-Gly-3M3SH peptidase activity using a coupled DTNB assay. Activity was indirectly measured through the spectrophotometric detection of TNB at 412 nm. Fractions 15 and 16 (black dotted lines) proceeded to the next purification step.


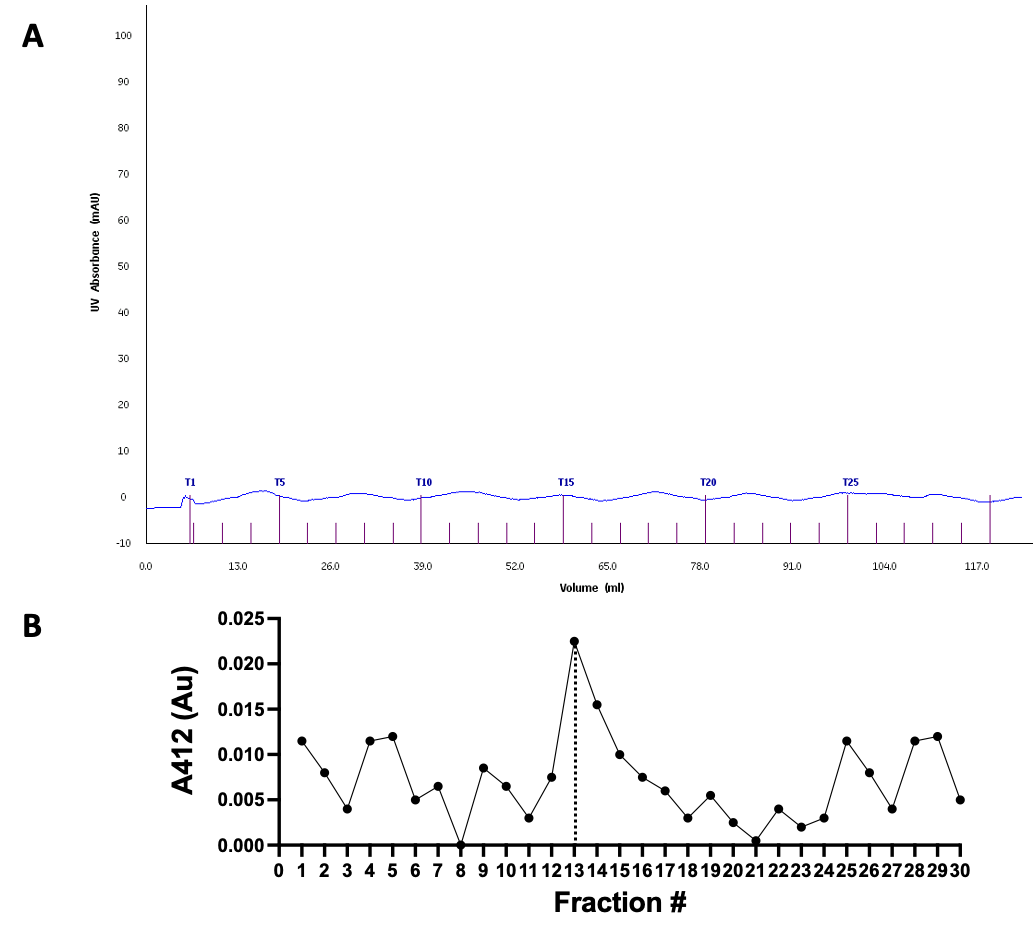


**Supplementary Data S5**. (A) Absorbance at 280 nm measured during size exclusion chromatography with the Sephacryl S200 resin (Cytiva). Elution fractions are labelled in red on the X-axis. (B) Elution fractions were tested for Cys-Gly-3M3SH peptidase activity using a coupled DTNB assay. Activity was indirectly measured through the spectrophotometric detection of TNB at 412 nm. Fraction 13 (black dotted lines) was analysed for the identification of the protein.

**Supplementary Data S6**. Cys-Gly-3M3SH dipeptidase activity is detected in SEC elution fractions 12-14 with fraction 13 having the highest signal. Samples from the elution fractions were tested neat (black dots) or concentrated by 133X (pink squares) in the coupled DTNB assay. Proteins were incubated with 1 mM Cys-Gly-3M3SH and 1 μM ShPatB for 30 minutes in the presence of DTNB. Dipeptidase activity was indirectly measured through the spectrophotometric detection of TNB at 412 nm.

**
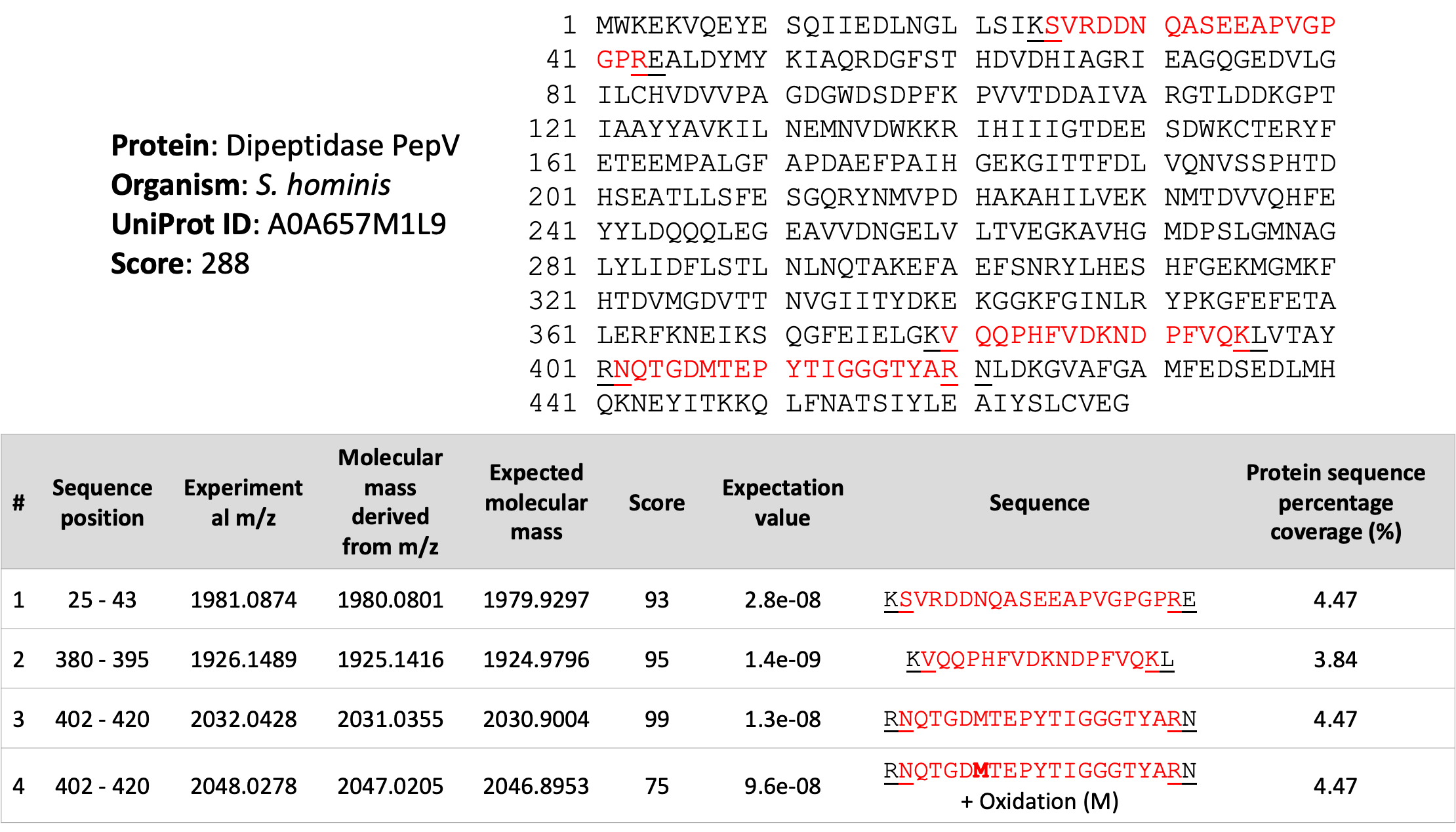
**

**Supplementary Data S7**. Identification of PepV by peptide mass fingerprinting using trypsin digest and MALDI-MS/MS. The total protein score is derived from the ions scores for each distinct sequence. No other proteins were matched with the experimentally isolated protein fragments.


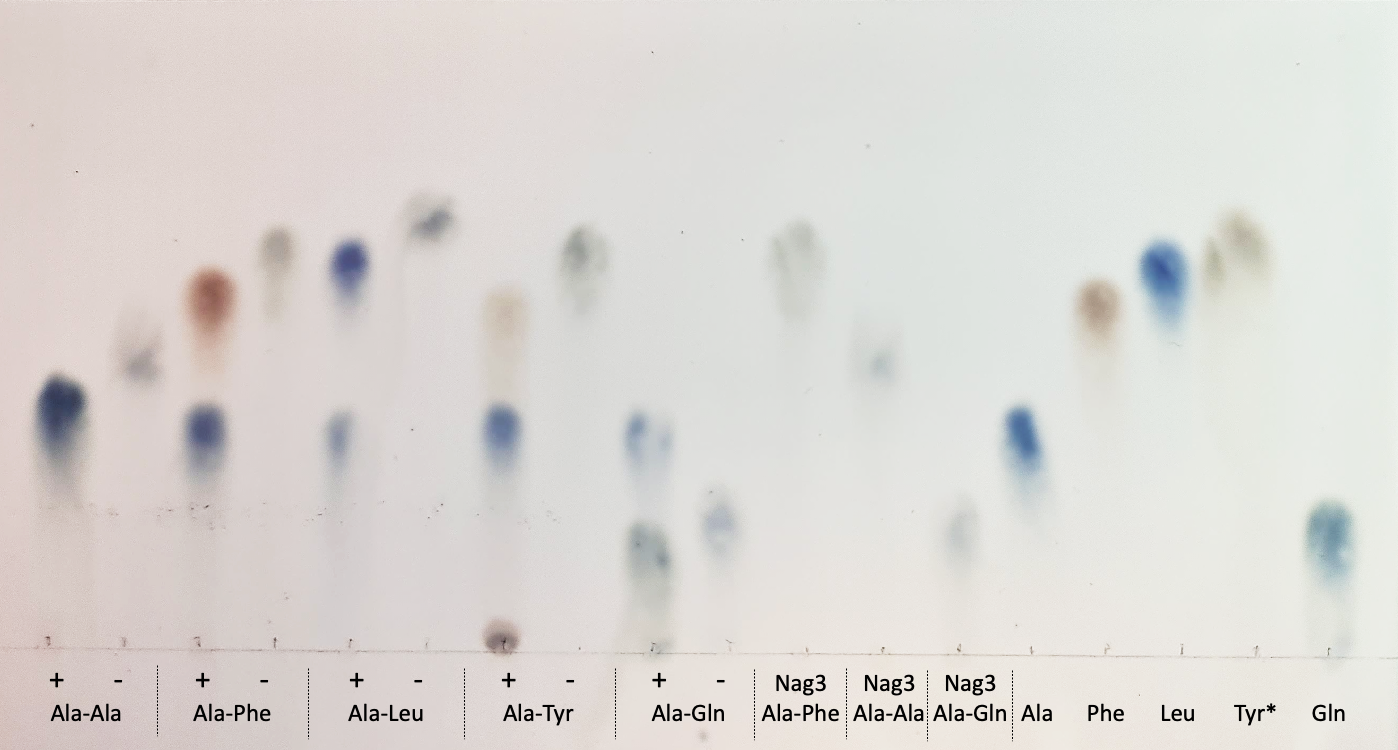


**Supplementary Data S9.** TLC assay ShPepV against a range of dipeptides. Peptides were incubated with (+) or without (-) PepV or the control enzyme Nag3 as indicated. Relevant peptide and amino acid controls were included. *L-Tyrosine was solubilised in 1M HCl. All other substrates and controls were solubilised in dH_2_O.


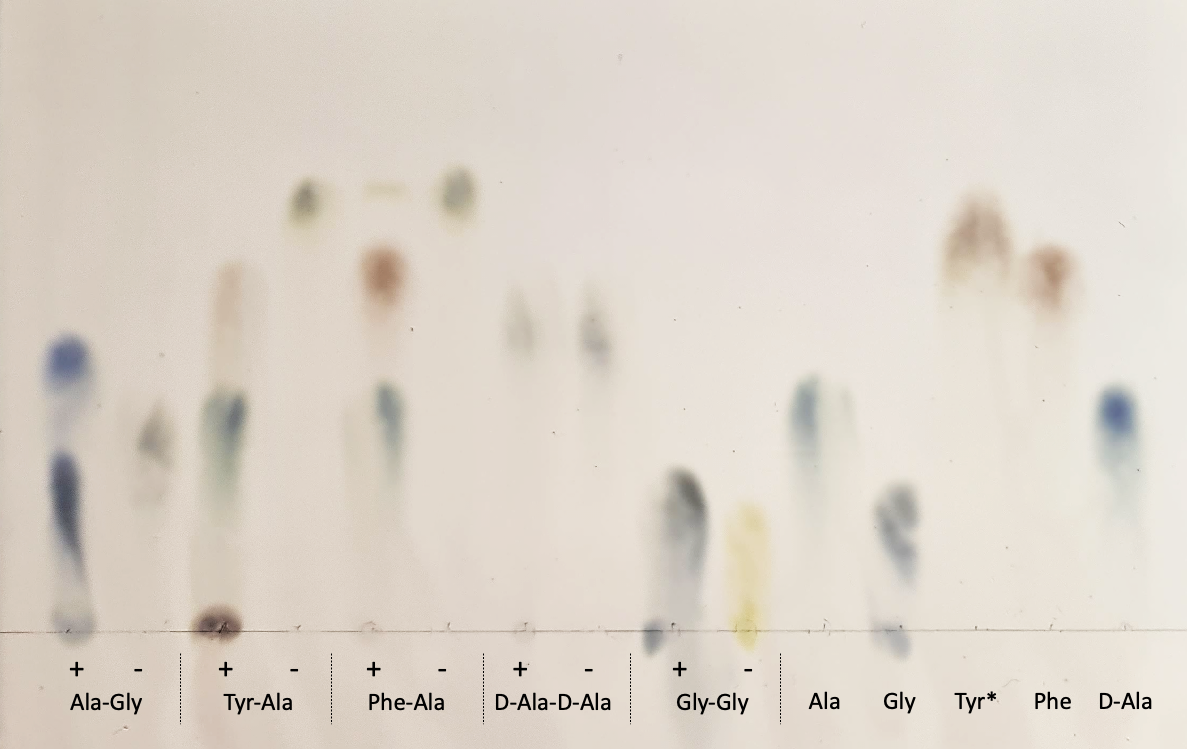


**Supplementary Data S10.** TLC assay ShPepV against a range of dipeptides. Peptides were incubated with (+) or without (-) PepV. Relevant peptide and amino acid controls were included. *L-Tyrosine was solubilised in 1M HCl. All other substrates and controls were solubilised in dH_2_O.


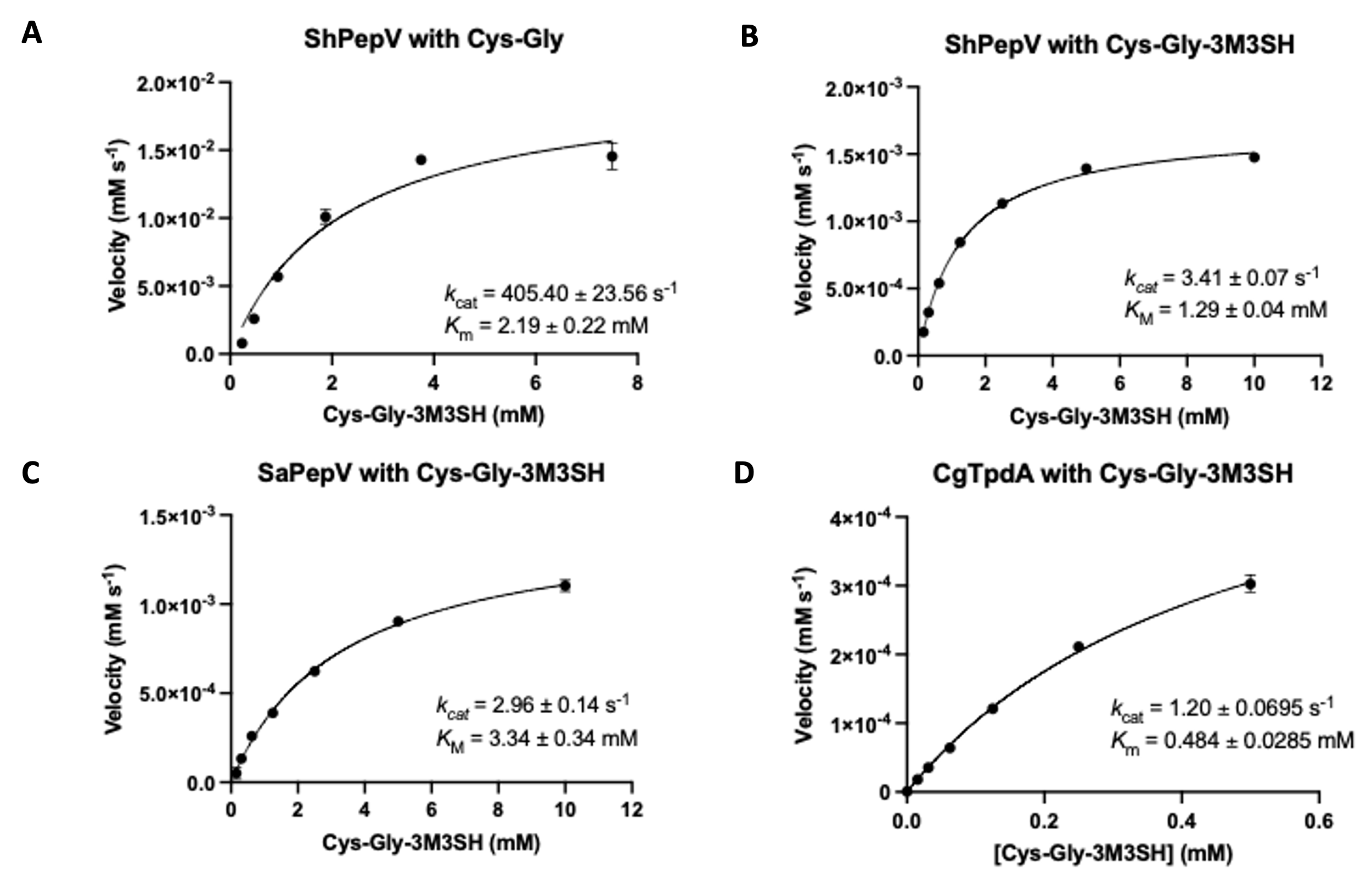


**Supplementary Data** **S11.** Michaelis-Menten kinetics of ShPepV with (A) Cys-Gly, (B) Cys-Gly-3M3SH and (C) SaPepV and (D) CgTpdA with Cys-Gly-3M3SH


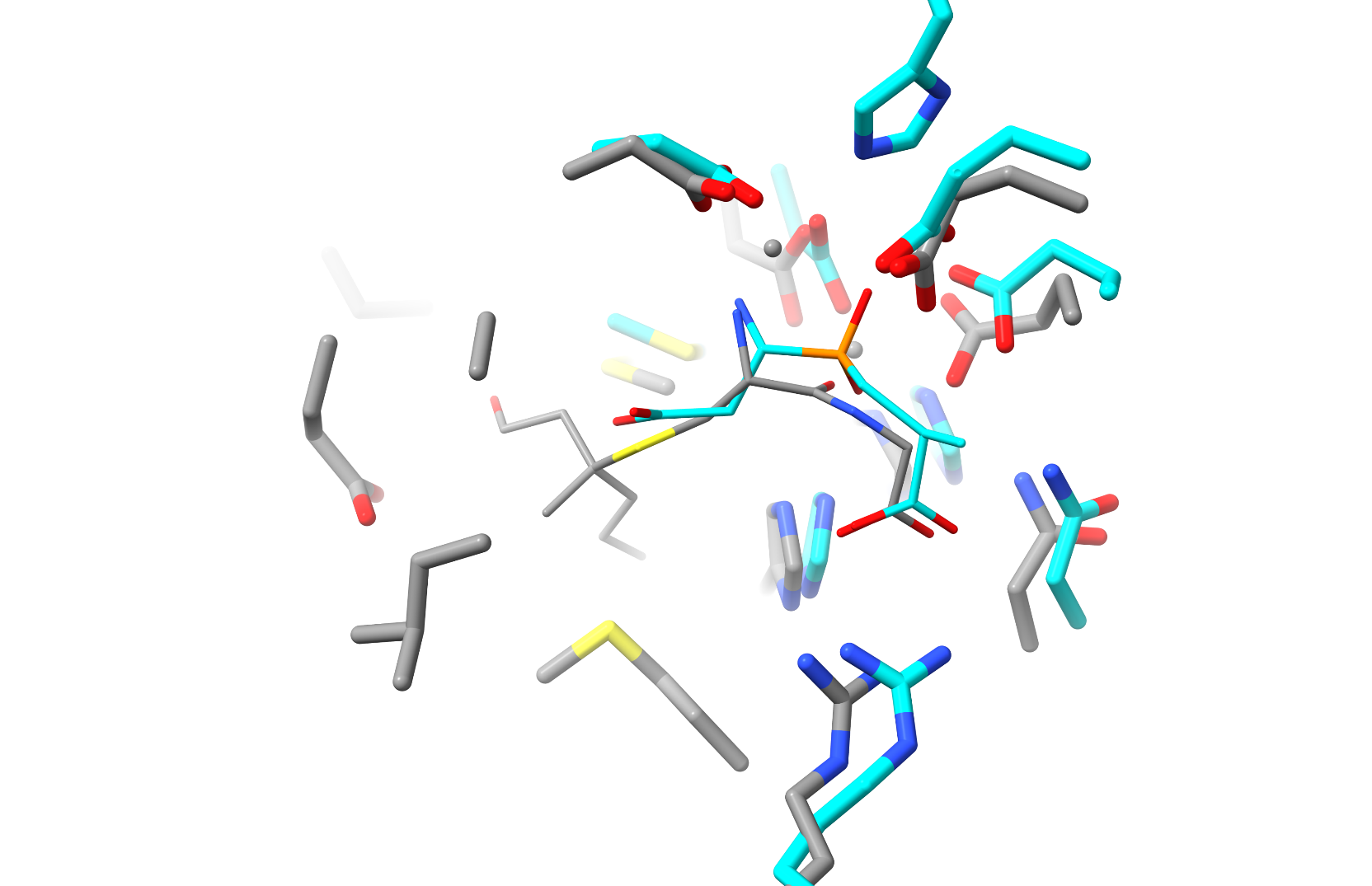


**Supplementary Data** **S12.** Overlay of the binding sites of ShPepV with Cys-Gly-3M3SH (Grey) and LdPepV with an Asp-Ala phosphinic acid inhibitor (Blue). Ligands are represented in thin cylinder form while amino acid residues are represented in thick cylinder form. Metals are represented by grey balls.


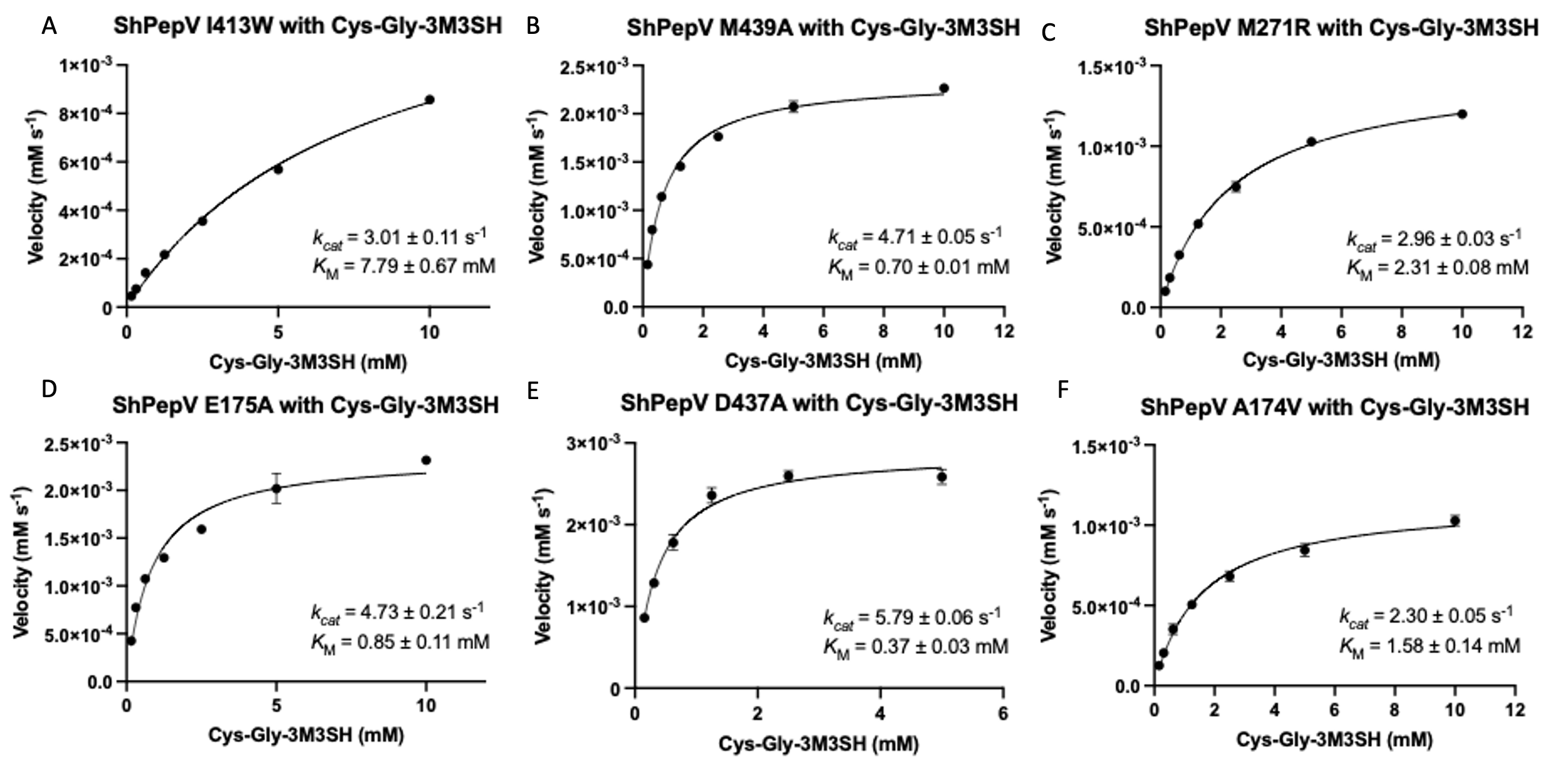


**Supplementary Data S13.** Michaelis-Menten kinetics of ShPepV mutants (A) I413W, (B) M439A, (C) M271R, (D) E175A, (E) D437A and (F) A174V with Cys-Gly-3M3SH


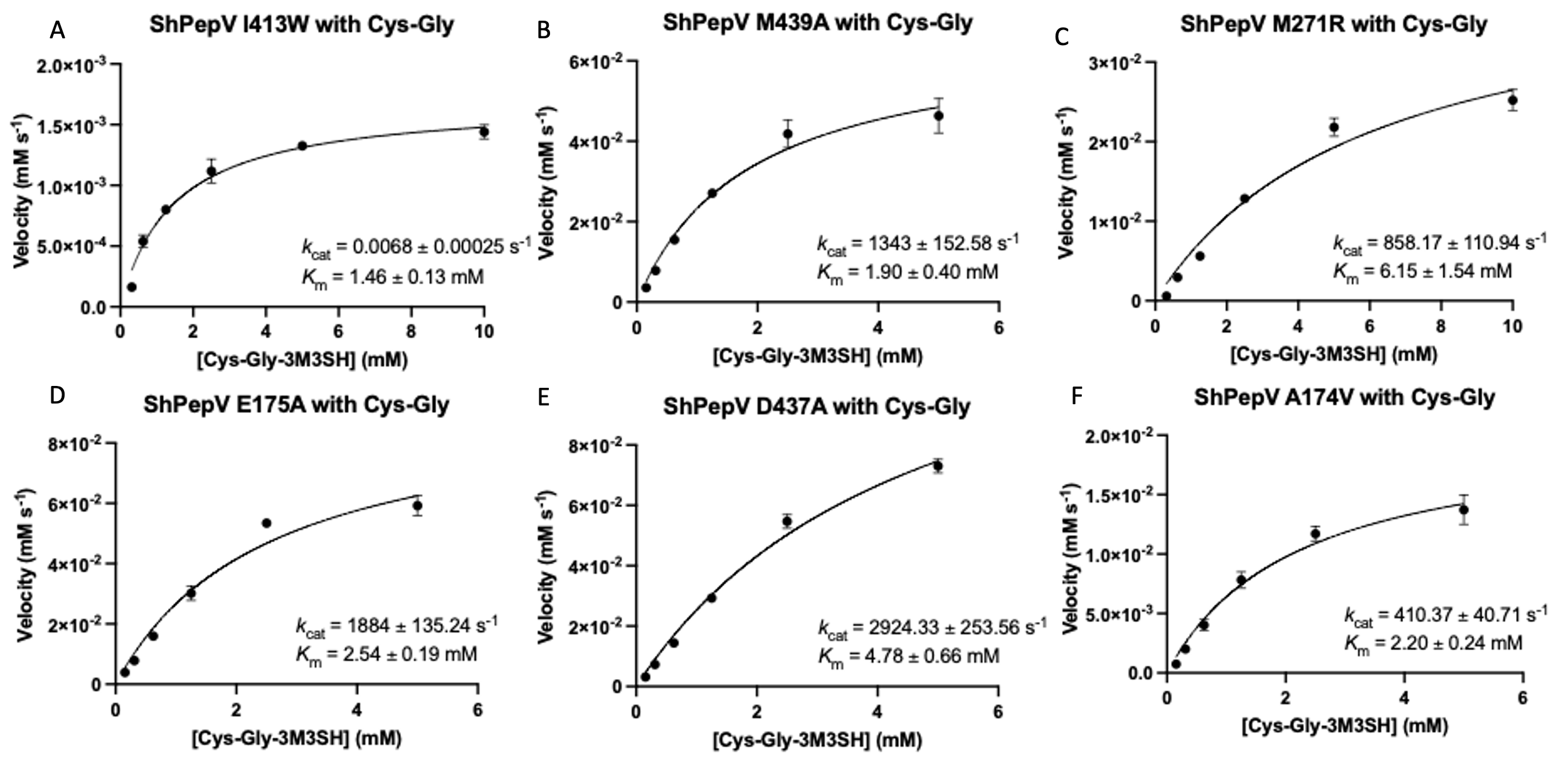


**Supplementary Data S14.** Michaelis-Menten kinetics of ShPepV mutants (A) I413W, (B) M439A, (C) M271R, (D) E175A, (E) D437A and (F) A174V with Cys-Gly.

**Supplementary Table 1**. Bacterial strains used in this study.


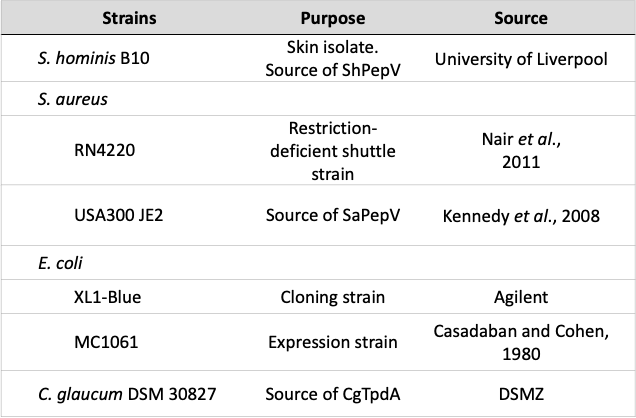


**Supplementary Table 2**. Plasmids used in this study.


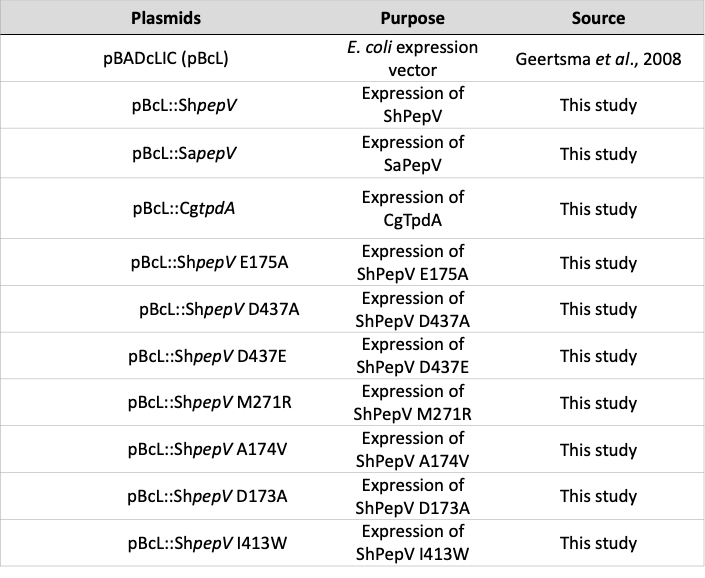


**Supplementary Table 3**. Primers used in this study


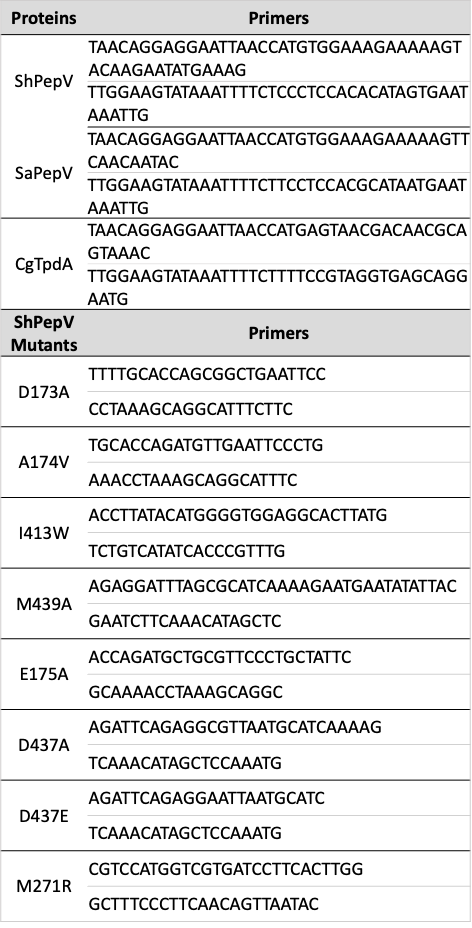


**Supplementary Table 4**. List of dipeptides and controls used for the characterisation of the peptidase activity of ShPepV. All non-glycine amino acids are in the L- form unless indicated. Activity of PepV against the di-/tripeptides are also included.


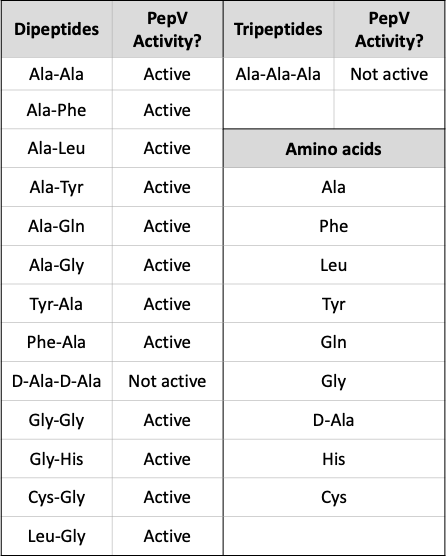


**Supplementary Table 5.** Kinetics of ShPepV mutants with Cys-Gly determined using a Cd-ninhydrin method. The fold change in catalytic efficiencies ((*k_cat_/K*_M_)_mut_/(*k_cat_/K*_M_)_WT_) and differences in transition state binding energies (ΔΔG^≠^) of the mutants against that of the wildtype (WT) protein were also calculated. (N.D.: not detectable)


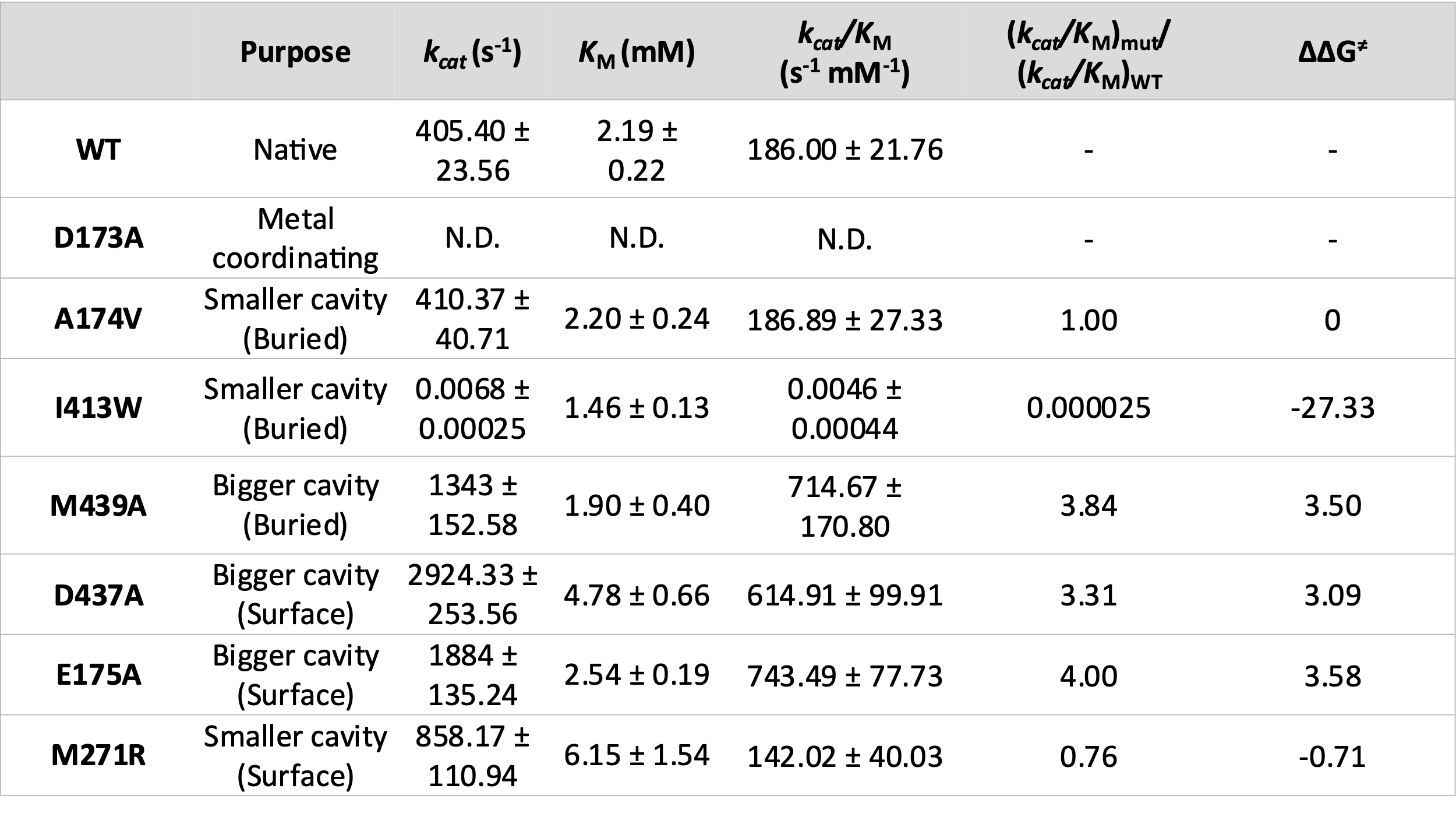

Supplement: Supporting Information [file mmc1.docx]
